# Loss of Gut Barrier Integrity In Lupus

**DOI:** 10.3389/fimmu.2022.919792

**Published:** 2022-06-20

**Authors:** Longhuan Ma, Laurence Morel

**Affiliations:** Department of Pathology, Immunology, and Laboratory Medicine, University of Florida, Gainesville, FL, United States

**Keywords:** gut barrier integrity, microbiota, dysbiosis, lupus, autoimmunity

## Abstract

Systemic Lupus Erythematosus is a complex autoimmune disease and its etiology remains unknown. Increased gut permeability has been reported in lupus patients, yet whether it promotes or results from lupus progression is unclear. Recent studies indicate that an impaired intestinal barrier allows the translocation of bacteria and bacterial components into systemic organs, increasing immune cell activation and autoantibody generation. Indeed, induced gut leakage in a mouse model of lupus enhanced disease characteristics, including the production of anti-dsDNA antibody, serum IL-6 as well as cell apoptosis. Gut microbiota dysbiosis has been suggested to be one of the factors that decreases gut barrier integrity by outgrowing harmful bacteria and their products, or by perturbation of gut immune homeostasis, which in turn affects gut barrier integrity. The restoration of microbial balance eliminates gut leakage in mice, further confirming the role of microbiota in maintaining gut barrier integrity. In this review, we discuss recent advances on the association between microbiota dysbiosis and leaky gut, as well as their influences on the progression of lupus. The modifications on host microbiota and gut integrity may offer insights into the development of new lupus treatment.

## Introduction

Systemic lupus erythematosus (SLE) is an autoimmune disease characterized by autoantibodies attacking multiple organs, including kidneys, joints, lungs, the heart, and the brain (1). Without known etiology, the cause of SLE has been attributed to a combination of genetic, epigenetic and environmental factors. Genome-wide association studies (GWAS) have identified around 180 lupus susceptibility loci in the human genome (2), most of which belong to three biological processes: signal transduction in lymphocytes, toll-like receptor signaling and type 1 interferon (IFN) production, and apoptotic cell processing (3). Dysregulated epigenetic factors also contribute to the development of SLE. Studies suggest that a global DNA hypomethylation exists in the CD4^+^ T cells and B cells of SLE patients (4), including genes involved in type 1 IFN signaling (5). Environmental factors can trigger SLE and cause flares in patients. Ultraviolet light, air pollution, infections and exposure to heavy metals are the most common triggers that can influence lupus progression by modulating epigenetic factors, affecting host immune status, increasing oxidative stress, regulating hormone levels or changing the configuration of the host microbiome (6). Increasing evidence supports that an unbalanced gut microbiota is associated with lupus pathogenesis (7–9). Dietary intervention modulating the composition of the gut microbiota, such as butyrate, tryptophan or resistant starch reversed some lupus phenotypes in murine models (10–12). It has been suggested that gut microbial dysbiosis enhance the inflammatory status and cause damage on the gut barrier, resulting in a “leaky gut” (13, 14). Gut permeability-mediated translocation of bacteria and their products into the systemic circulation could activate the immune system and promote autoimmunity in genetically predisposed populations (15). Conversely, an increased immune response would also break the gut homeostasis resulting in microbial dysbiosis and increased gut permeability (16). In this review, we summarize the findings linking the loss of gut barrier integrity to lupus and evaluate the mechanisms responsible for leaky gut in this disease as well as its contribution to SLE pathogenesis.

## Evidence of Gut Dysbiosis and Leaky Gut in SLE

### Gut Dysbiosis in Lupus

Over 1000 bacterial species have been identified in the human gut with around 160 species presenting in each individual microbiota (17) and most of them are belong to four dominant bacterial phyla, as Bacteroidetes, Firmicutes, Proteobacteria, and Actinobacteria (18). The composition of gut microbiome is highly variable. Genetic factors, such as histocompatibility complex (MHC) polymorphism, and environmental factors, including ethnicity, diet and geography, have been correlated to the gut microbiota structure (19–21). A meta-analysis of studies comparing the fecal microbiome of SLE patients and healthy controls showed a lower diversity in SLE patients with a lower abundance of *Ruminococcaceae* (9). SLE patients with active lupus disease showed less diverse gut microbiota but a significantly higher abundance of the bacterial phylum Proteobacteria (22). Within the altered microbiota of SLE patients with disease active, disease activity was positively correlated with the abundance of the genera *Streptococcus, Campylobacter, Veillonella*, and negatively correlated with the abundance of *Bifidobacterium* (23). A lower ratio of Firmicutes to Bacteroidetes was observed in SLE patients with a reduction of some families in the Firmicutes phylum (24, 25). However, various bacterial families of Firmicutes, including *Lactobacill*, *Clostridiaceae* and *Lachnospiraceae*, have been reported to have a greater abundance in multiple mouse models of lupus (11, 12, 22). However, the MRL/lpr lupus prone mice showed a decreased abundance of *Lactobacilli* and the addition of *Lactobacillus* spp. improved disease outcomes in this model (26). Similarly, segmented filamentous bacteria (SFB), bacteria belonging to the phylum Firmicutes, expand in the gut of B6SKG mice that develop a lupus-like phenotype and they promote the development of lupus by increasing Th17 cell differentiation (27).

**Figure 1:**
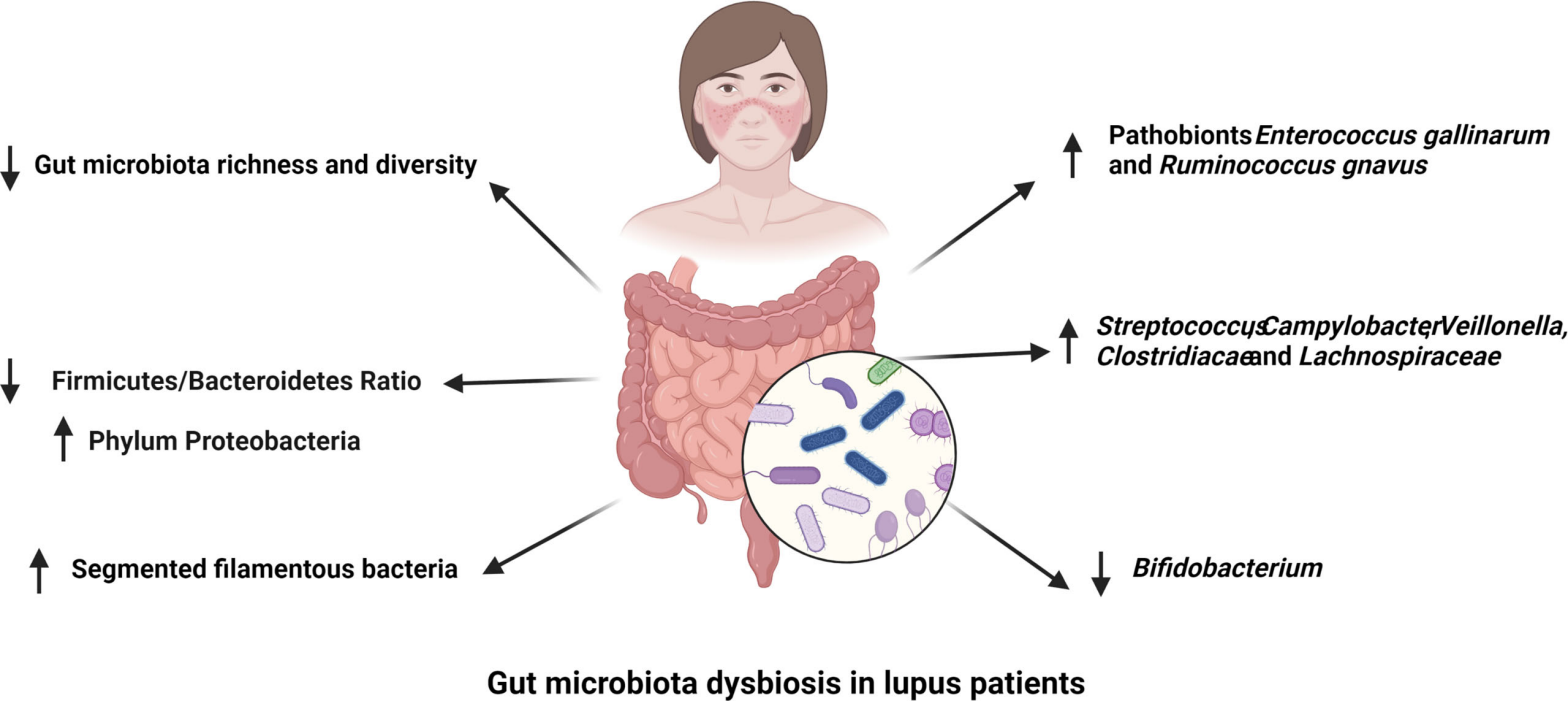
Gut microbiota dysbiosis in lupus patients. The richness and diversity of gut microbiota as well as the Firmicutes to Bacteroidetes ratio are decreased in different cohorts of lupus patients. Segmented filamentous bacteria have been reported to have a higher abundance in the gut microbiota of lupus patients. The abundance of *Streptococcus*, *Campylobacter*, *Veillonella*, *Clostridiacae* and *Lachnospiraceae* are positively correlated to SLE disease activity, while that of *Bifidobacterium* is negatively associated with lupus activity. Finally, pathobionts *Ruminococcus gnavus* and *Enterococcus gallinarum* are enriched in the gut of lupus patients.

Overall, SLE patients with active disease showed a distinct dysbiosis in the gut microbiota. In comparison, lupus patients in remission had a comparable microbial diversity with healthy control subjects despite of a lower Firmicutes/Bacteroidetes ratio (23, 24). The abundance of bacteria in the phylum Firmicutes and genus *Bifidobacterium* are negatively correlated with SLEDAI score in lupus patients, while bacteria from genus *Streptococcus* are positively correlated with lupus activity in different human lupus cohorts (23, 28, 29). At species level, *Ruminococcus gnavus* (*R. gnavus)* was substantially enriched in patients with lupus nephritis and the presence of antibodies against a specific strain of *R. gnavus* was associated with disease activity and the level of anti-dsDNA antibodies (30, 31). This finding is significant because it was reported in two different cohorts of lupus patients despite the large genetic and environmental variability inherent to human populations. There is therefore strong evidence from multiple studies that SLE is associated with gut dysbiosis (**Figure 1**), and results obtained with mouse models support a contribution of the altered microbiome to SLE pathogenesis, with the identification of several potential pathobionts.

### Leaky Gut in Lupus

Mounting evidence suggests that a leaky gut is presented by some, if not all, SLE patients (7, 8). The detection of microbial components in blood stream of lupus patients suggests the penetration of microorganisms and their products into systemic circulation may be mediated by an increased intestinal permeability (32–34). The recovery of bacterial DNA from the liver of lupus patients with autoimmune hepatitis supported the translocation of *Enterococcus gallinarum* (*E. gallinarum*) into systemic organs (35). Besides complete bacteria, various bacterial components have been detected in the blood of SLE patients, implicating their leaking out of the gut. SLE patients and their first-degree relatives showed a higher level of lipopolysaccharide (LPS) or endotoxin, or expression of genes induced by these bacterial products, than healthy controls (32, 33). Additionally, (1 ​→ ​3)-β-D-glucan, a component of fungal cell walls, was detected in the serum of patients with active lupus nephritis (34). Soluble CD14 and α1-acid glycoprotein, two serum biomarkers for microbial translocation, have also been detected at a higher level in SLE patients than healthy controls (8, 36). In the other direction, the detection of serum proteins, such as albumin and calprotectin, in the feces further supports a loss of barrier integrity in the gut in SLE patients (30, 35).

The presence of endotoxin in the blood of MRL/lpr lupus-prone mice also suggested gut barrier dysfunction, which was further supported by a FITC-dextran assay, in which fluorescent dextran is gavaged and its presence is measured in the serum (37). Interestingly, supplementation with a commensal bacterium, *Lactobacillus reuteri* (*L. reuteri*), rescued these phenotypes in these mice, suggesting that microbiota play a role in maintaining gut barrier integrity. However, the expansion of *L. reuteri* has been reported in spontaneous *Tlr7* transgenic and inducible TLR7 lupus-prone mouse models (12). An increased gut permeability in FITC-dextran assay, and the translocation of *Lactobacillus* spp. to internal organs, including mesenteric lymph node (MLN), liver, and spleen, in TLR7-dependent mouse models suggest that *L. reuteri* may have different effects on different genetic backgrounds, with the potential involvement of TLR7 signaling. The presence of endotoxin in the plasma as well as a FITC-dextran assay indicated an increased gut permeability in lupus-prone NZBWF1 mice (38). In addition, the detection of *E. gallinarum* in the MLN and liver of (NZW × BXSB)F_1_ lupus-prone mice, suggest that complete bacteria can be translocated into systemic organs through a leaky gut (35). However, there was no evidence of leaky gut in the B6.*Sle1.Sle2.Sle3* lupus-prone mice in spite of gut dysbiosis that was sufficient to transfer some autoimmune phenotypes (11). Overall, an impaired gut barrier was detected in SLE patients and multiple, but not all, lupus murine models suggesting causal but not obligate links between the two.

## Relationship Between Gut Dysbiosis and Leaky Gut in SLE

### Microbial Dysbiosis Promotes Intestinal Permeability

Gut dysbiosis combined with increased intestinal permeability has been reported in various diseases or disorders (39), implying possible causal relationships between these two factors. Little is known about what causes a loss of gut barrier integrity in SLE, except that *E. gallinarium*, a pathobiont that is expanded in (NZW × BXSB)F_1_ lupus-prone mice, translocates in monocolonized non-autoimmune gnotobiotic (or germfree, GF) mice, suggesting an intrinsic ability to disrupt the gut epithelial barrier (35). Results obtained in other disease models may be however indicative of some of the mechanisms by which dysbiosis may promote a leaky gut in SLE.

Intestinal permeability, microbial dysbiosis as well as age-associated inflammation develop in old non-autoimmune mice. The transfer of fecal microbiota from old mice into GF young mice increased gut permeability in recipient mice, suggesting that age-associated changes in microbiome composition can promote intestinal permeability (40). The young recipients colonized with microbiota from old mice also had a higher level of plasma TNFα, implying that microbial dysbiosis can also induce age-associated inflammation, which may further exacerbate leaky gut in the old mice. On the other hand, *Tnfa*-deficiency improved gut dysbiosis in old mice, suggesting that host immunity can affect the microbiome configuration as well.

Loss of certain bacteria in the gut may lead to an impaired gut barrier function. Obese and diabetic mice show increased intestinal permeability, metabolic endotoxemia and a low-grade inflammation. A mixture of prebiotics specifically increased the abundance of *Bifidobacterium* spp., which improved systemic and hepatic inflammation, intestinal integrity and endotoxemia. The findings suggest that modifications on host microbiota affect the host immune status and gut integrity (41). *In-vitro* stimulation of intestinal epithelial cells with TLR-2 ligands induced the redistribution of tight junction proteins, resulting in an improved monolayer integrity. Since TLR2 is highly expressed by intestinal epithelia cells *in vivo* where it recognizes bacterial components, such as diacylated or tritylated lipopeptides, the absence of the producing bacteria may affect gut integrity (42). Indeed, feeding mice with lipoteichoic acid (LTA), a ligand for TLR2, increased mucin expression and reduced inflammation and gut leakage (43). In this sense, supplementation with beneficial bacteria may restore the gut barrier function. *Lactobacillus plantarum* increases the expression of tight junction proteins, including ZO-1 and Occludin, in humans. Similarly, exposing a Caco-2 cells monolayer to *L. plantarum* enhanced intestinal integrity *via* the translocation of ZO-1 protein to tight junctions. However, when TLR2 was blocked by neutralizing antibodies, the protective effect was eliminated, suggesting that *L. plantarum* may confer its protection by activating TLR2 (44). When an anti-inflammatory molecule generated by *Faecalibacterium prausnitzii* was supplemented in a type 2 diabetes mellitus model, it restored gut barrier function and increased ZO-1 expression, suggesting that it directly contribute to gut barrier integrity (45). Similarly, extracellular vesicles secreted by *Akkermansia muciniphila* were found at a higher level in the feces of healthy subjects compared to type II diabetes patients, and oral delivery of these extracellular vesicles increased gut barrier integrity in mice (46). Another commensal bacterium, *Lactobacillus salivarius*, showed a capacity to restore barrier function in a monolayer of epithelial cells. The study demonstrated that *L. salivarius* ameliorated the disassembly and relocation of tight junctions induced by H_2_O_2_ in Caco-2 cell monolayers, leading to an improved barrier integrity (47). In graft-versus-host disease (GVHD) patients, oral administration of *Bacteroides fragilis* increased the levels of short chain fatty acids (SCFAs) and IL-22, as well as the number of regulatory T cells, which may account for the improved tight junction integrity and reduced inflammation (48). The findings demonstrate that specific bacteria may improve leaky gut, which may imply that leaky gut was caused by their loss.

Outgrowths of commensal pathobiont and/or pathogenic infections can also be harmful to gut integrity. For example, an enrichment of *Bacteroides* and *Prevotellaceae*_UGG-001 was detected in a mouse model of experimental autoimmune hepatitis (EAH). Administration of broad-spectrum antibiotics prior to EAH induction prevented the development of hepatitis and increased gut integrity (49). The commensal bacteria *Bacteroides fragilis* (*B. fragilis*) showed a capacity to modulate the development of colitis (50). Mice colonized with enterotoxigenic *B. fragilis* showed an increased intestinal permeability with damaged epithelial E-cadherin (51), which was mostly likely mediated by the tight junction degrading metalloprotease toxin produced by *B. fragilis* (52). Besides *B. fragilis*, there are other pathogens expressing gut damaging toxins, including toxin A and B producing *Clostridium difficile* (53), enterotoxin producing *Clostridium perfringens* (54), cytotoxic necrotizing factor 1 producing *E.coli* (55), vacuolating toxin producing *Helicobacter pylori* (56), internalin producing *Listeria monocytogenes* (57) and Zonula occludens toxin producing *Vibrio cholerae* (58). Moreover, enteric *Pseudomonas fluorescens* can induce the secretion of zonulin, a negative modulator of tight junctions, resulting in cytoskeleton changes and tight junction disassembly in a cell line (59). Additionally, infection with the protozoans *Giardia intestinalis* and *Blastocystis hominis* increased intestinal permeability in mice (60). Rotavirus can also increase the permeability of gut barrier by altering the location of tight junction protein occludin (61). Thus, many microorganisms can affect positively or negatively gut barrier integrity, and their identification in the context of lupus may be critical to restore gut barrier function in SLE patients.

### Leaky Gut Exacerbates Gut Dysbiosis

Damaged gut barrier integrity can active the innate immune system resulting in the recruitment of various immune cells at the site of injury. The cytokines, enzymes and growth factors secreted by these immune cells disturb the immune homeostasis and induce inflammation (62). An inflamed microenvironment in the gut allows the bloom of some bacteria, such as *Enterobacteriaceae* (63). In addition, a leaky gut allows the passage of bacterial components and even living bacteria into host systemic circulation (7, 8), inducing innate and adaptive immune responses, which break the balance of tolerance and immunity in the gut leading to a dysbiotic microbiome (16). Induced gut leakage by dextran sulfate solution (DSS) in multiple mouse models have demonstrated that gut leakage can enhance systemic inflammation and alter host microbiome composition (64, 65). Leaky gut may also allow the undigested food particles to travel out of the gut lumen and get into blood stream. As external antigens, these food particles may provoke strong immune responses leading to gut dysbiosis (39).

### Genetic Variants Associated With Leaky Gut

An GWAS for inflammatory bowel disease (IBD) has identified susceptibility genes that are associated with intestinal barrier function, which included genes involved in mucus and glycoprotein regulation (*ECM1*, *MUC3A* and *MUC19*), membrane receptor kinase (*ERRFI1*), membrane transport (*ITLN1* and *VDR*), tight junction regulation (*PTPN2*), epithelial restitution (*PTGER4*), cell polarity (*PARD3*), cell adhesion (*CDH1* and *LAMB1*), tight junction assembly (*GNA12*, *MAGI2*, *MYO9B* and *CDH1*) and epithelial differentiation (*HNF4A*) (66). A Crohn’s disease risk locus, Chr 5p13.1, regulates the expression of the prostaglandin receptor EP4, which is expressed in intestinal epithelial cells and affects gut barrier function (67). The mutation on this gene could highly increase the risk for leaky gut. In addition, a *CARD15* polymorphism has been associated with enhanced gut permeability and the development of Crohn’s disease (68). Gluten-derived peptide gliadin disrupts gut integrity by rearranging actin and tight junction proteins in celiac disease (69–71). The human leukocyte antigen (HLA) DQ2 and/or DQ8 is required for the presentation of gliadin to T cells and consequent increased inflammatory responses, suggesting a causative role of these two genetic factors in the gut permeability induction (72).

Contrary to these intestinal autoimmune diseases, none of the many genetic variants that have been associated with lupus susceptibility are directly linked to barrier integrity, except possibility for SLC17A4 (73). SLC17A4 is an organic anion transporter expressed in the gut, and it is expressed at a high level in gnotobiotic mice as compared to mice housed in conventional conditions (74). It is therefore possible that SLC17A4 variants are associated with SLE susceptibility through their regulation of gut integrity in response to bacterial signals. However, bacterial translocation has been detected in *Tlr7* Tg mice as well as in mice treated with TLR7 agonist (12). *Tlr7* polymorphisms and copy number have been associated with lupus susceptibility (75), and it would be of great interest to evaluate whether these genetic variations are associated with leaky gut and/or gut dysbiosis. Collectively, gene variants related to tight junction proteins and some aspects of immune response may work as major susceptibility factors for leaky gut, although no evidence for such genes has yet been found in SLE.

## Mechanisms Leading to the Loss of Barrier Integrity in Lupus

### Immune Activation and Inflammation

**Figure 2:**
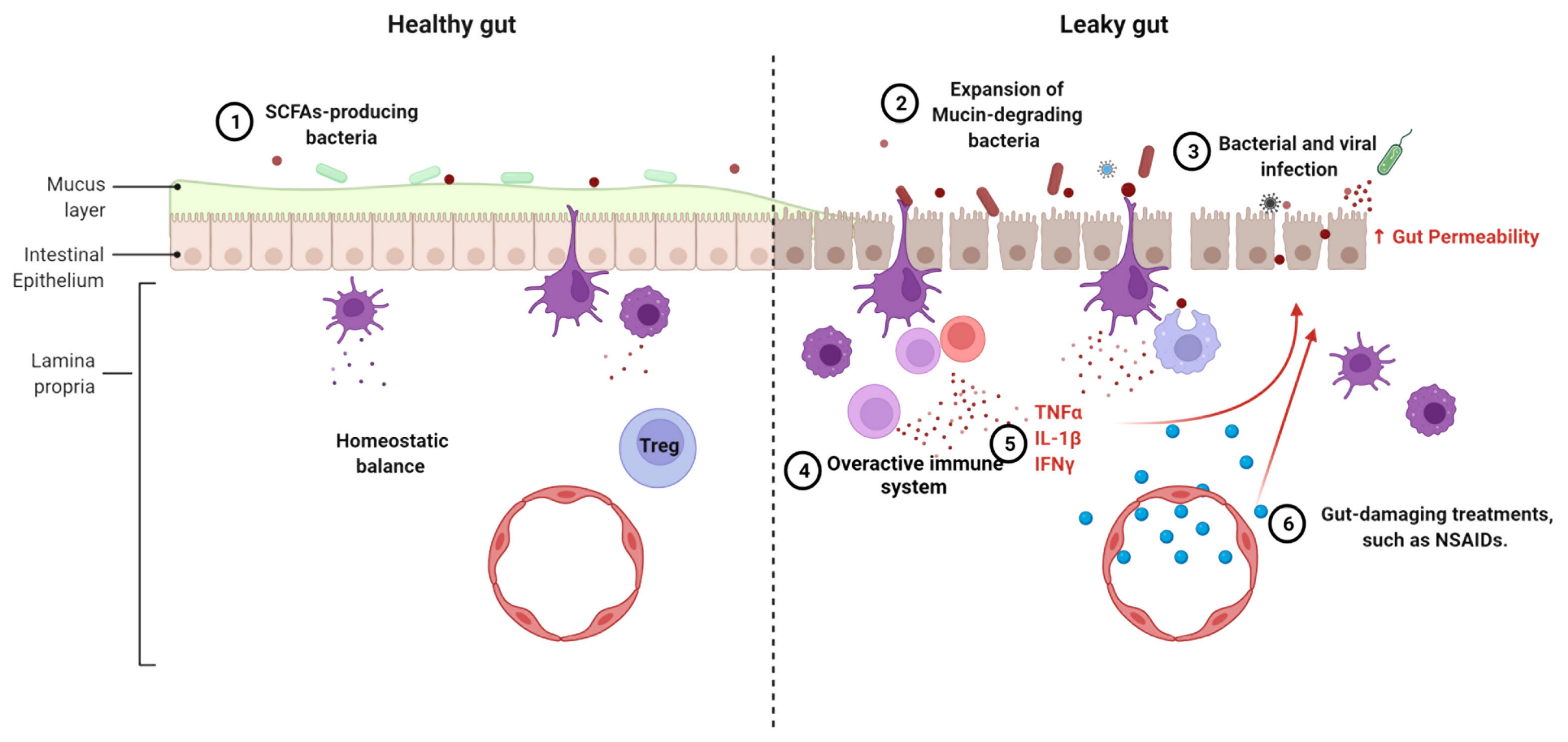
Mechanisms leading to the loss of gut barrier integrity in lupus. The following mechanisms can compromise a healthy gut (left) leading to a leaky gut (right), singly or in combination, 1. The reduction or even loss of SCFA-producing bacteria is linked to alterations of the mucus layer lining the gut. 2. Expansion of mucin-degrading bacteria, such *as R. gnavus*, promotes gut leakage. 3. Frequent intestinal infections induce inflammation and tissue damage. 4. The overactive immune system leads to chronic inflammation and recruitment of inflammatory cells. 5. Proinflammatory cytokines affect gut integrity. 6. Some lupus treatments cause damage on gut barrier function.

The chronic inflammation that characterizes lupus has been attributed to dysfunctional B cells, T cells and dendritic cells (76). In addition, an altered expression of inflammatory cytokines and cell surface receptors has been identified in monocytes and macrophages from SLE patients (77, 78). Various cytokines and chemokines play a role in maintaining gut epithelial cell integrity. For example, IL-13 and CXCL10 can modulate the movement of gut epithelial cells to expel parasites to avoid parasitic infection (79). Proinflammatory cytokines TNF-*α*, IL-1β and IFN-γ, all of which have been implicated in SLE pathogenesis (80–82), suppress the expression of tight junction proteins, alter the arrangement of tight junctions, and modulate the actin cytoskeleton in intestinal epithelial cells, resulting in a compromised gut barrier (83, 84). Furthermore, IL-1β and IFN-γ exert their functions on tight junctions through the activation of the NF-κB pathway (85, 86). An increased NF-κB activation has been associated with lupus pathogenesis, including through the lower expression of its negative regulator A20 (87). However, NF-κB is required for epithelial cell replacement and mice lacking NF-κB showed a lower expression of antimicrobial peptides and an increased apoptosis in enterocytes (88). The different effects of NF-κB pathway on gut barrier integrity suggest that its activation may be key for the maintenance of immune homeostasis in gut epithelial tissues. Taken together, the altered immune state in lupus patients may destroy the balance between immunity and tolerance in the gut, causing damage to the intestinal epithelial cells (**Figure 2**).

### Gut Dysbiosis Induced by Lupus

In lupus patients, the dysregulated immune state in the intestine may induce microbial dysbiosis which in turn affects the integrity of gut (8). An enrichment of commensals that damage gut integrity has been detected in SLE patients and mouse models, such as *R. gnavus* and *E. gallinarium* (28, 30). The combination of a defective immunity and the use of immunosuppressive drugs lead to a high frequency of pathogenic infections in SLE patients, including with *Staphylococcus aureus*, *Salmonella enterica*, *Escherichia coli*, *Streptococcus pneumonia* and *Mycobacterial* species (89). *Salmonella typhimurium* showed a capacity to increase gut permeability by upregulating the expression of Claudin-2, a leaky gut mediator (90). It is therefore possible that *Salmonella* blooms occurring in SLE patients may also promote leaky gut through this mechanism. Translocation of oral microbes to the gut has been reported in lupus patients (23, 30). Accordingly, inoculation of mice with *Porphyromonas gingivalis* or *Fusobacterium nucleatum*, two common oral bacteria, induced gut barrier damage and aberrant inflammation (91, 92). Beside bacteria, various viral infections, including HIV, CMV, bacteriophages and dengue virus, can also cause increased gut permeability leading to a leaky gut (93–95). The relationship between viral infections and leaky gut in lupus has however not been investigated.

In accordance with gut dysbiosis, an aberrant fecal microbial metabolism was detected in SLE patients (23). Several fecal metabolites have shown potential to modulate gut homeostasis. Short chain fatty acids (SCFA) produced by some bacteria promote the proliferation of regulatory T (Treg) cells and suppress the production of inflammatory cytokines (96). Besides this well-documented modulating effect on inflammation, SCFA improve intestinal barrier function (97, 98). A low concentration of the SCFA butyrate decreased monolayer permeability and enhanced transepithelial resistance in epithelial cells *in vitro*, while a high concentration of butyrate had the inverse effect. Mechanistically, butyrate decreases permeability by accelerating the assembly of tight junctions *via* the activation AMPK (99, 100). *Bifidobacterium* species increase gut barrier integrity by producing acetate, which increased the expression of the tight junction gene *Occludin* (101). Since most butyrate-producing bacteria belong to the Firmicutes phylum, inflammation and impaired gut barrier integrity may be induced by the decreased relative abundance of Firmicutes in SLE patients. In the *Tlr7* Tg model of lupus, dietary resistant starch reduced the translocation of *L. reuteri* by inducing the production of SCFA (7). This implied, at least in this model, a direct role of SCFA in maintaining gut barrier integrity to prevent the translocation of pathobionts that amplify lupus pathogenesis (**Figure 2**). Our group has recently identified an aberrant tryptophan metabolism in the gut of the B6.*Sle1.Sle2.Sle3* mice that is largely regulated by the microbiota (102). Reducing dietary tryptophan ameliorated autoimmune phenotypes with an increased Treg suppressive function and lower autoantibody titers (11). One of tryptophan metabolites produced by bacteria, indole, can improve gut barrier function by upregulating the expression of tight junction proteins in the gut (103, 104). An altered tryptophan metabolism is also found in SLE patients (102) and it may contribute to impaired gut integrity, although this has never been formally investigated.

### Side Effects of SLE Treatments

The medications used to treat SLE patients induce a leaky gut directly or as the result of infections induced by immunosuppression. Besides antibiotics, many non-antibiotic medicines have shown adverse effects on host intestinal homeostasis, including proton pump inhibitors (PPIs), metformin, No-steroidal anti-inflammatory drugs (NSAIDs), opioids and antipsychotics (105, 106). Specifically, NSAIDs and hydroxychloroquine, which are widely used to treat SLE, have gastro-intestinal side effects (107–109). NSAIDs can induce mitochondrial and endoplasmic reticulum damage and oxidative stress in intestinal epithelial cells, enhancing gut permeability and local inflammation (110). Treatment of SLE patients with hydroxychloroquine reduced the abundance of *Enterobacteriaceae*, and glucocorticoids, a group of drugs broadly used to treat lupus, reduced microbial diversity (9). The calcineurin inhibitor tacrolimus (FK 506) increased gut permeability in humans and rats through inhibiting mitochondrial respiration in gut epithelial cells (111). Voclosporin (Lupkynis) is a calcineurin inhibitor that has been recently approved for the treatment of lupus nephritis (112), and it would be of interest to monitor its potential effect of gut permeability. Finally, treatment with immunomodulatory drugs increase the risks for infection with bacteria and viruses in SLE patients (113–115). As mentioned above, intestinal infections induced by these treatments may trigger immune activation and inflammation in the hosts leading to damage to the gut barrier (**Figure 2**).

## Leaky Gut Exacerbates Lupus Progression

**Figure 3:**
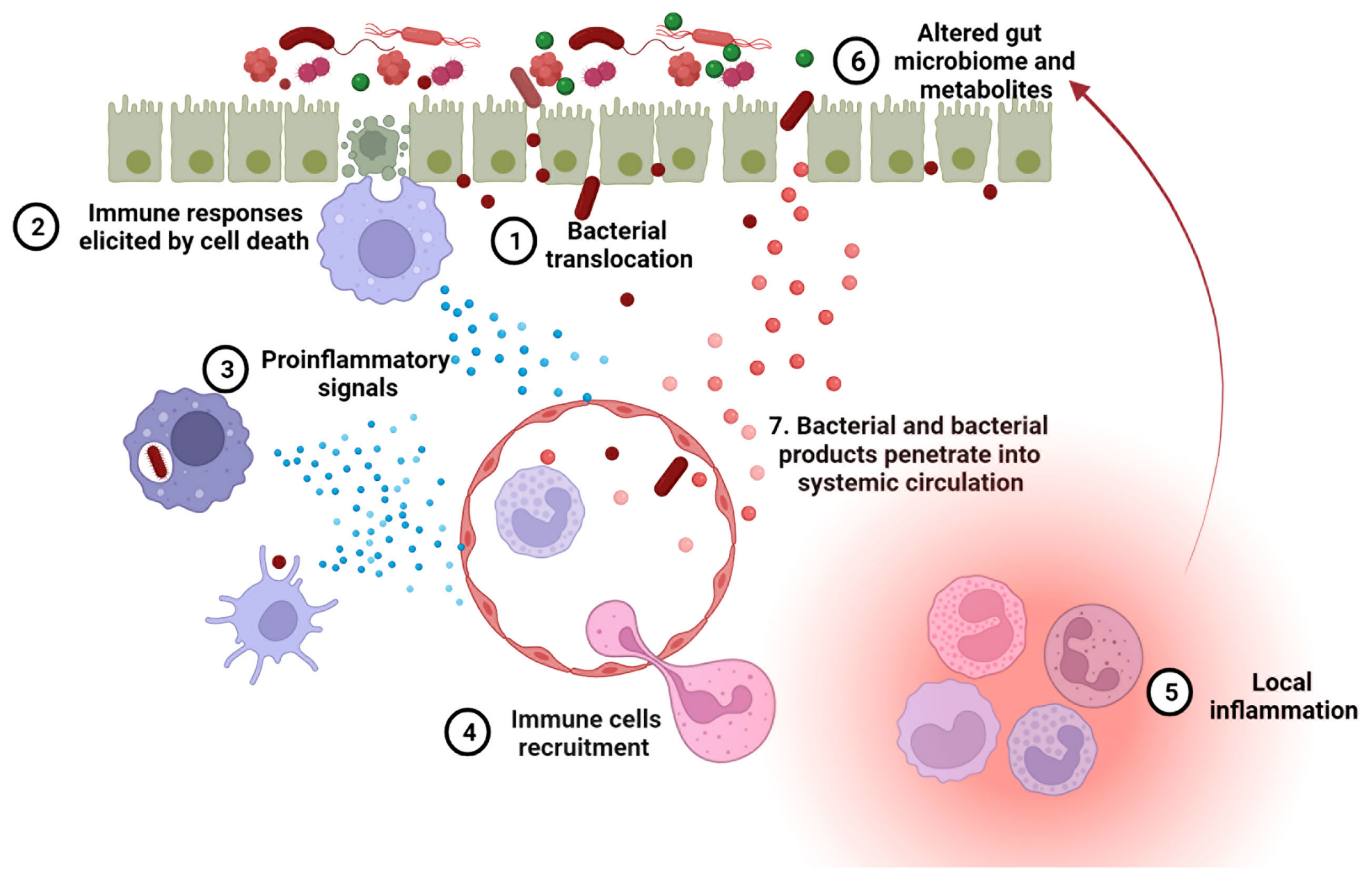
A leaky gut exacerbates lupus progression. 1. Leaky gut-mediated bacterial translocation has been linked to the initiation of autoimmune responses. 2. Increased enterocyte cell death in leaky gut activates innate immune cells, inducing an overactive immune system. 3. The phagocytosis of invading bacteria by innate immune cells promotes inflammation. 4. Upon pro-inflammatory signaling, more immune cells are recruited to the leaky gut area. 5. Immune cell focal aggregation triggers local inflammation. 6. Enhanced local inflammation causes gut microbial dysbiosis and dysregulated fecal metabolites, both of which are related to lupus progression. 7. Bacteria and bacterial products can translocate into the blood stream through impaired blood vessels and then induce autoimmunity in systemic organs.

A leaky gut allows foreign antigens to pass into the systemic circulation, which may provoke both local and systemic immune responses. As mentioned above, LPS and (1 ​→ ​3)-β-D-glucan have been detected in the sera of SLE patients (32, 64). These two components are known to mediate secretion of proinflammatory cytokines, including type I IFN, through activation of TLR-4 and dectin-1, respectively (116). When given systemically to mice, lipoteichoic acid, a major cell wall component from gram-positive bacteria, induces various autoimmune diseases, including SLE (117, 118). The bacterial amyloid curli and DNA form immunogenic complexes that accelerate the progression of SLE, *via* the generation of autoantibodies and type I IFN responses (119). An impaired gut barrier may allow the translocation of such antigens from intestinal lumen into internal environment, resulting in a higher-level autoimmune response. Exposure of *E. gallinarum* to human hepatocytes induced the generation of autoimmune-promoting factors, such as β2GPI and type I IFN, and activated the AHR pathway (35). Thus, the translocation of pathobiont *E. gallinarum* into livers of patients with SLE and autoimmune hepatitis may contribute to the development of autoimmune disease in these patients, as it did in mice. Collectively, microorganisms and their products passing through an impaired gut barrier into internal environment can result in a hyperactive immune system in hosts contributing to lupus progression (**Figure 3**).

The contribution of leaky gut to lupus pathogenesis was directly assessed with a chemically induced injury with DSS in the spontaneous FcGRIIb^−/−^ model and the pristane induced model. The detection of bacteria in the MLN as well as endotoxin and (1 ​→ ​3)-β-D-glucan in the blood of these mice showed that the DSS treatment mediated the translocation of gut microorganisms and their products into systemic circulation. Importantly, DSS increased systemic inflammation, such as IL-6 production, as well as renal pathology. Additionally, an enhanced apoptosis was observed in the MLN and spleen, which may explain the increased presence of anti-dsDNA antibodies in the blood and immune complex deposits in the kidney (64). Taken together, these results suggest that a leaky gut may exacerbate the severity of lupus pathogenesis.

## Therapeutic Strategies to Restore Gut Barrier Function

### Lifestyle Modifications

Diet has been known to affect the gut integrity directly or indirectly by modifying the host microbiota (120). Studies suggest that a high-fat diet can directly downregulate the expression and distribution of tight junction proteins (121–123) and induce the secretion of bile acid into the gut lumen increasing gut permeability (123–125). A combination of low fiber and high fat content increased the abundance of mucin degrading bacteria in the gut (126, 127), which may compromise the mucus layer in the lumen and increase the susceptibility to leaky gut. Moreover, a high fat diet changes the ratio of Bacteroidetes/Firmicutes in adult C57BL/6J mice (128), may partly explain its harmful impacts on gut homeostasis as Bacteroidetes are commonly associated with chronic intestinal inflammation, while many of beneficial bacteria belong to the Firmicutes phylum. Notably, high fat diet exacerbates lupus phenotypes in TLR8-deficient lupus-prone mice. The effects are attributed to an enhanced TLR7 signaling in dendritic cells (129). As mentioned above, an upregulated TLR7 signaling increase gut permeability, therefore, high fat diet may cause more severe gut leakiness in people genetically predisposed to lupus. In addition, a diet rich in animal proteins but not in plant proteins exacerbates intestinal inflammation in a chronic colitis model by increasing the proinflammatory response of monocytes (130). Similarly, a high-glucose and high-fructose diet can also induce inflammation in the gut leading to the alteration of tight junction proteins, which increased gut permeability (131). As patients with SLE have a high prevalence of metabolic syndrome featuring glucose tolerance, hypertriglyceridemia and others, the disturbed glucose and lipid metabolism in lupus patients may further aggravate diet-mediated gut permeability (132). Additionally, food additives present in numerous processed food items have shown adverse impacts on host microbiota. An increased consumption of processed food and additives used by food industry may explain the increased gut dysbiosis and gut leakage in the general population as well as lupus patients (133).

On the other hand, certain dietary components or supplements can restore gut integrity. Various nutrient components, including vitamin D, have shown potentials to improve gut integrity (120). However, vitamin D deficiency is highly prevalent in patients with SLE (134–136). The supplementation of vitamin D may restore the gut barrier function in lupus patients. Administration of retinoic acid (RA), a major oxidative metabolite of vitamin A, increases the barrier function of epithelial cells *in vitro* and the relative abundance of *Lactobacillus* spp., a group of bacteria that support gut barrier function in mice, suggesting that retinoic acid can directly enhance gut integrity or through modifying host microbiota (137). In a similar way, all-*trans*-retinoic acid (tRA) treatment on pristine-induced lupus mouse model reversed gut leakage, showing a reduced serum endotoxin level. At same time, tRA treatment also modified microbiome composition which was dysregulated by pristine injection, supporting a dual role of retinoic acid in affecting gut barrier function and microbiome configuration. Supplementation of glutamine in children improved intestinal barrier function (138). In contrast, a low level of glutamine was reported in SLE patients (139, 140), which may also account for an impaired gut barrier in lupus patients. In addition, as mentioned above, SCFA can promote gut integrity by enhancing tight junction assembly (100). Supplementation of resistant starch, a highly fermentable fiber, in *Tlr*7 Tg mouse model rescued the gut leakiness (12). Overall, these results suggest that a healthy diet or dietary modifications that improve lupus outcomes may also improve gut barrier integrity, although it has never been tested in SLE patients.

The association of psychological stress and intestinal dysbiosis and permeability have been established (141, 142). Multiple lines of evidence support that stress and depression can change the gut microbiota composition through hormones levels, gut motility and inflammation, indirectly influencing gut permeability (143). When chronic depression was induced in a mouse model, increased corticotropin-releasing hormone, serotonin level and gut motility was observed in the hosts and these changed parameters may explain altered microbial profile in the gut (142). Acute psychological stress was demonstrated to active mast cell in the gut by increased corticotropin-releasing hormone. The induced overactive immunity increased permeability in small intestine in humans (141). People living with lupus are likely to feel anxiety and even hopeless because of the uncontrolled disease activity. The changes in physical appearance also bring pressures in social interactions (144). All these psychological factors may play roles in increasing gut permeability in lupus patients.

### Medical Treatment

Larazotide acetate (LA) or AT1001, a tight junction regulator, inhibited the redistribution and rearrangement of tight junction components induced by zonula occludens toxin and its eukaryotic analogue zonulin, maintaining monolayer integrity of IEC6 and Caco-2 cells (145). Treatment with LA on a mouse model of celiac disease blocked gliadin-induced gut inflammation and permeability (145). LA is currently tested in phase III clinical trials as an adjunct therapeutic to enhance intestinal barrier function in celiac disease patients (146). Moreover, a zonulin neutralizing antibody showed a similar protective function as LA on lung permeability in two mouse models of acute lung injury (147). As increased serum zonulin levels has been correlated to intestinal permeability in several autoimmune diseases (148), blockage of zonulin pathway may be a potential therapeutic strategy to restore the gut barrier function. More specifically to lupus, a high level of zonulin was detected in the fecal samples of SLE patients (Preprint) (149). Oral administration of LA reversed gut permeability in C57BL/6 mice colonized with a strain of *R. gnavus* derived from lupus patients (Preprint) (150).These findings suggest that LA provides a promising therapeutic option to improve gut barrier functions in lupus.

Some drugs to treat diabetes, hypertension and other diseases have shown a potential to modify gut integrity. Metformin is widely used in type 2 diabetes to lower blood glucose level. Administration of metformin induced gut microbial dysbiosis with an increased relative abundance of the gut opportunistic pathogen *Escherichia_Shigella* (151). However, an opposite effect was observed in a sepsis-related liver injury (SLI) rat model. Administration of metformin in aged SLI rats decreased the abundance of *Klebsiella* and *Escherichia_Shigella* and increased that of *Bifidobacterium*, *Muribaculaceae*, *Parabacteroides distasonis* and *Alloprevitella*. Fecal microbiota transfers (FMT) from metformin treated SLI rats decreased liver damage, colon barrier dysfunction as well as inflammation in recipient SLI rats (152). Metformin also decreased inflammation and gut leakage in obese mice fed with a high-fat diet by modifying their gut microbiome and increasing goblet cell proliferation leading to a higher mucus production (153). A similar microbiota restorative function of metformin was also found in a model of high-fat diet induced type 2 diabetes (154). These results suggest the potential of metformin in treating host microbial dysbiosis and leaky gut. Further research is required to investigate underlying mechanisms, and to understand why the outcomes of a treatment with metformin on gut microbiome and barrier integrity may be context dependent. Metformin has shown beneficial effects in mouse models of lupus (155) as well as in SLE patients (156), at least in part through the normalization of T cell metabolism (155, 157). It would be of great interest to investigate whether metformin also restores gut microbial homeostasis and barrier integrity. Another diabetes drug, berberine, also showed beneficial effects on epithelial integrity. In the Caco-2 cell monolayer model of intestinal barrier, berberine prevented IFNγ and TNFα-induced gut permeability by inhibiting myosin light chain kinase-dependent phosphorylation of the myosin light chain mediated by HIF-1α (158). Berberine treatment did not increase the expression of tight junction proteins in another Caco-2 cell assay but increased their transepithelial electrical resistance. This finding led the hypothesis that berberine does not affect the expression and distribution of tight junctions but tightens the tight junction integrity (159). Although berberine and its derivatives have not been tested in lupus, they are being evaluated in numerous inflammatory conditions, including those involving inflammatory T cells such as lupus (160). The concomitant assessment of their effect on gut barrier integrity may reveal valuable mechanistic insights.

Captopril, a hypertension drug that works as angiotensin-converting enzyme inhibitor, has shown long-lasting beneficial effects on the microbial composition, permeability and pathology of the gut in spontaneously hypertensive rats (161). β-blockers, another class of blood-pressure reducing drugs, alleviated intestinal permeability and decreased bacterial translocation in patients with portal hypertension (162). Since a leaky gut accelerates the pathogenesis of SLE, medication that restore gut integrity may serve as efficacious therapeutic agents for lupus.

Finally, vaccination with heat-killed gut pathobiont *E. gallinarum* reversed gut permeability and bacterial translocation induced by this bacterium, as well as decreased the production of autoantibodies and prolonged the survival of lupus-prone mice (35). Taken together, a number of therapeutic interventions have proved protective effects on gut integrity in various diseases and models. The application of these treatment options to lupus patients warrants further research to determine their clinical efficacy.

### Antibiotics and Probiotics

As described above, infections or enrichment of harmful bacteria in the gut could increase intestinal permeability, therefore antibiotics-mediated selective elimination may restore the gut barrier function. Indeed, the gastrointestinal antibiotic rifaximin decreased the abundance of *Clostridium* and improved intestinal barrier function in a mouse model of chronic stress (163). Similarly, the rifaximin treatment of patients with decompensated cirrhosis decreased the abundance of *Streptococcus* in the gut and ameliorated endotoxemia with alleviated gut permeability (164). Since SLE disease activity has been positively correlated with the abundance of *Streptococcus* in the gut (23) and infections of *Clostridium difficile* was also linked to the mortality of SLE patients (165), the suppressing effects of rifaximin on both bacteria may offer a new approach in the treatment of lupus. Furthermore, rifaximin prevents stress-induced mucosal inflammation and intestinal barrier impairment in rats by increasing the expression of occludin (166). Oral supplementation with two other non-absorbable antibiotics, neomycin and polymyxin B, improved intestinal leakiness in Western diet or high salt diet mouse models (167, 168). The findings suggest that antibiotics can ameliorate microbiota dysbiosis triggered by environmental factors as well as local inflammation, leading to a healthier gut. In lupus prone MRL/lpr mice, an antibiotic cocktail or vancomycin alone restored the microbiome structure and gut barrier function, supporting a therapeutic benefit of antibiotics in lupus (37). However, since a lower microbiome diversity and richness have been reported to SLE patients (22, 25, 30, 31, 169), it could be further reduced by broad-spectrum antibiotics, which should therefore be used with caution. More sophisticated interventions are needed to target specific pathogens without killing beneficial bacteria in SLE patients.

Probiotics are living commensal or nonpathogenic microorganisms that provide health benefits to hosts (170). A number of studies have shown that supplementation of probiotics can exclude invading bacterial pathogens by suppressing their adhesion and by producing antimicrobial compounds (171). As summarized above, an increased infection rate has been reported in lupus patients due to abnormalities in their immune system and immunosuppressive treatments. Thus, probiotics may serve as a substitute for antibiotics to prevent bacterial infections and improve infection-mediated gut permeability. Many probiotics exert their functions through immune regulation, particularly through the modification of immune cell populations and the balance between pro- and anti-inflammatory cytokines (172). Administration of the probiotics *Lactobacillus rhamnosus* and *L. delbrueckii* to pristane-induced lupus mice reduced the populations of Th1 and Th17 cells and the levels of proinflammatory cytokines IFNγ and IL-17 (173). Furthermore, supplementation of NZB/W F1 mice with specific strains of *L. reuteri* suppressed the MAP kinase and NF-*κ*B signaling pathways, reducing the levels of IL-1β, IL-6 and TNFα (174). Because of the gut-damaging role of multiple pro-inflammatory cytokines, including IL-1β, TNFα and IFNγ (83, 84), suppression on proinflammatory cytokines mediated by probiotics may contribute to an improved gut barrier as well. Lastly, probiotics can promote the expression of mucus glycoprotein and strengthen tight junctions, therefore enhancing the integrity of gut barrier (175). Notably, *Lactobacillus* treatment on lupus prone MRL/lpr mice significantly increased the expression of multiple tight junction proteins, including ZO1, Occludin, and Claudin-1, in intestinal epithelial cells, suggesting that certain probiotics may rescue gut permeability detected in SLE patients and restore the normal gut barrier function (176).

## Conclusion

In summary, increased gut permeability, or leaky gut, in lupus patients can be induced by overactive immune responses, gut microbial dysbiosis, side effects of treatments, or any combination of these factors (**Figure 2**). The resulting gut leakage allows bacteria to escape from the gut lumen and enter the systemic circulation, which in turn promotes local and systemic inflammation, changing gut microbiome configuration and metabolites profile. Overall, the leaky gut, the overactive immune responses and microbial dysbiosis can exacerbate each other, creating a vicious feed-forward loop (**Figure 3**). Certain lifestyle modifications, medications, antibiotics and probiotics have shown promising results on improving gut permeability in other diseases or mouse models. Considering the close relationships between immunity, microbiome and intestinal integrity in lupus patients, an unbiased evaluation of these leaky gut interventions should be evaluated in pre-clinical models of the disease for their ability to restore the balance in the immune system and/or gut microbiome, and ultimately, to change disease outcomes.

## Author Contributions

LMa and LMo wrote the review. All authors contributed to the article and approved the submitted version.

## Funding

This publication is supported by grant RO1 AI143313 from the NIH to LMo.

## Conflict of Interest

The authors declare that the research was conducted in the absence of any commercial or financial relationships that could be construed as a potential conflict of interest.

## Publisher’s Note

All claims expressed in this article are solely those of the authors and do not necessarily represent those of their affiliated organizations, or those of the publisher, the editors and the reviewers. Any product that may be evaluated in this article, or claim that may be made by its manufacturer, is not guaranteed or endorsed by the publisher.

## References

[1] Tsokos GC. Systemic Lupus Erythematosus. N Engl J Med (2011) 365(22):2110–21. doi: 10.1056/NEJMra1100359 22129255

[2] Ha E Bae SC Kim K. Large-Scale Meta-Analysis Across East Asian and European Populations Updated Genetic Architecture and Variant-Driven Biology of Rheumatoid Arthritis, Identifying 11 Novel Susceptibility Loci. Ann Rheum Dis (2021) 80(5):558–65. doi: 10.1136/annrheumdis-2020-219065 PMC8053349 33310728

[3] Harley IT Kaufman KM Langefeld CD Harley JB Kelly JA. Genetic Susceptibility to SLE: New Insights From Fine Mapping and Genome-Wide Association Studies. Nat Rev Genet (2009) 10(5):285–90. doi: 10.1038/nrg2571 PMC2737697 19337289

[4] Patel DR Richardson BC. Epigenetic Mechanisms in Lupus. Curr Opin Rheumatol (2010) 22(5):478–82. doi: 10.1097/BOR.0b013e32833ae915 PMC3819175 20445453

[5] Absher DM Li X Waite LL Gibson A Roberts K Edberg J. Genome-Wide DNA Methylation Analysis of Systemic Lupus Erythematosus Reveals Persistent Hypomethylation of Interferon Genes and Compositional Changes to CD4+ T-Cell Populations. PLos Genet (2013) 9(8):e1003678. doi: 10.1371/journal.pgen.1003678 23950730 PMC3738443

[6] Barbhaiya M Costenbader KH. Environmental Exposures and the Development of Systemic Lupus Erythematosus. Curr Opin Rheumatol (2016) 28(5):497–505. doi: 10.1097/BOR.0000000000000318 27428889 PMC4965307

[7] Dehner C Fine R Kriegel MA. The Microbiome in Systemic Autoimmune Disease: Mechanistic Insights From Recent Studies. Curr Opin Rheumatol (2019) 31(2):201–7. doi: 10.1097/BOR.0000000000000574 PMC6408954 30624285

[8] Silverman GJ Azzouz DF Alekseyenko AV. Systemic Lupus Erythematosus and Dysbiosis in the Microbiome: Cause or Effect or Both? Curr Opin Immunol (2019) 61:80–5. doi: 10.1016/j.coi.2019.08.007 PMC6901743 31590039

[9] Xiang S Qu Y Qian S Wang R Wang Y Jin Y. Association Between Systemic Lupus Erythematosus and Disruption of Gut Microbiota: A Meta-Analysis. Lupus Sci Med (2022) 9(1):e000599. doi: 10.1136/lupus-2021-000599 35346981 PMC8961174

[10] He H Xu H Xu J Zhao H Lin Q Zhou Y. Sodium Butyrate Ameliorates Gut Microbiota Dysbiosis in Lupus-Like Mice. Front Nutr (2020) 7:604283. doi: 10.3389/fnut.2020.604283 33262998 PMC7688247

[11] Choi SC Brown J Gong M Ge Y Zadeh M Li W. Gut Microbiota Dysbiosis and Altered Tryptophan Catabolism Contribute to Autoimmunity in Lupus-Susceptible Mice. Sci Transl Med (2020) 12(551):eaax2220. doi: 10.1126/scitranslmed.aax2220 32641487 PMC7739186

[12] Zegarra-Ruiz DF El Beidaq A Iniguez AJ Lubrano Di Ricco M Manfredo Vieira S Ruff WE. A Diet-Sensitive Commensal Lactobacillus Strain Mediates TLR7-Dependent Systemic Autoimmunity. Cell Host Microbe (2019) 25(1):113–27.e6. doi: 10.1016/j.chom.2018.11.009 30581114 PMC6377154

[13] Lobionda S Sittipo P Kwon HY Lee YK. The Role of Gut Microbiota in Intestinal Inflammation With Respect to Diet and Extrinsic Stressors. Microorganisms (2019) 7(8):271. doi: 10.3390/microorganisms7080271 PMC6722800 31430948

[14] Shen ZH Zhu CX Quan YS Yang ZY Wu S Luo WW. Relationship Between Intestinal Microbiota and Ulcerative Colitis: Mechanisms and Clinical Application of Probiotics and Fecal Microbiota Transplantation. World J Gastroenterol (2018) 24(1):5–14. doi: 10.3748/wjg.v24.i1.5 29358877 PMC5757125

[15] Fine RL Manfredo Vieira S Gilmore MS Kriegel MA. Mechanisms and Consequences of Gut Commensal Translocation in Chronic Diseases. Gut Microbes (2020) 11(2):217–30. doi: 10.1080/19490976.2019.1629236 PMC7053960 31306081

[16] Levy M Kolodziejczyk AA Thaiss CA Elinav E. Dysbiosis and the Immune System. Nat Rev Immunol (2017) 17(4):219–32. doi: 10.1038/nri.2017.7 28260787

[17] Qin J Li R Raes J Arumugam M Burgdorf KS Manichanh C. A Human Gut Microbial Gene Catalogue Established by Metagenomic Sequencing. Nature (2010) 464(7285):59–65. doi: 10.1038/nature08821 20203603 PMC3779803

[18] Faith JJ Guruge JL Charbonneau M Subramanian S Seedorf H Goodman AL. The Long-Term Stability of the Human Gut Microbiota. Science (2013) 341(6141):1237439. doi: 10.1126/science.1237439 23828941 PMC3791589

[19] Kubinak JL Stephens WZ Soto R Petersen C Chiaro T Gogokhia L. MHC Variation Sculpts Individualized Microbial Communities That Control Susceptibility to Enteric Infection. Nat Commun (2015) 6:8642. doi: 10.1038/ncomms9642 26494419 PMC4621775

[20] Gaulke CA Sharpton TJ. The Influence of Ethnicity and Geography on Human Gut Microbiome Composition. Nat Med (2018) 24(10):1495–6. doi: 10.1038/s41591-018-0210-8 30275567

[21] Singh RK Chang HW Yan D Lee KM Ucmak D Wong K. Influence of Diet on the Gut Microbiome and Implications for Human Health. J Transl Med (2017) 15(1):73. doi: 10.1186/s12967-017-1175-y 28388917 PMC5385025

[22] Luo XM Edwards MR Mu Q Yu Y Vieson MD Reilly CM. Gut Microbiota in Human Systemic Lupus Erythematosus and a Mouse Model of Lupus. Appl Environ Microbiol (2018) 84(4):e02288–17. doi: 10.1128/AEM.02288-17 29196292 PMC5795066

[23] Li Y Wang HF Li X Li HX Zhang Q Zhou HW. Disordered Intestinal Microbes Are Associated With the Activity of Systemic Lupus Erythematosus. Clin Sci (Lond) (2019) 133(7):821–38. doi: 10.1042/CS20180841 30872359

[24] Hevia A Milani C Lopez P Cuervo A Arboleya S Duranti S. Intestinal Dysbiosis Associated With Systemic Lupus Erythematosus. mBio (2014) 5(5):e01548–14. doi: 10.1128/mBio.01548-14 PMC4196225 25271284

[25] He Z Shao T Li H Xie Z Wen C. Alterations of the Gut Microbiome in Chinese Patients With Systemic Lupus Erythematosus. Gut Pathog (2016) 8:64. doi: 10.1186/s13099-016-0146-9 27980687 PMC5146896

[26] Zhang H Liao X Sparks JB Luo XM. Dynamics of Gut Microbiota in Autoimmune Lupus. Appl Environ Microbiol (2014) 80(24):7551–60. doi: 10.1128/AEM.02676-14 PMC4249226 25261516

[27] Shirakashi M Maruya M Hirota K Tsuruyama T Matsuo T Watanabe R. Effect of Impaired T Cell Receptor Signaling on the Gut Microbiota in a Mouse Model of Systemic Autoimmunity. Arthritis Rheumatol (2022) 74(4):641–53. doi: 10.1002/art.42016 34725966

[28] He J Chan T Hong X Zheng F Zhu C Yin L. Microbiome and Metabolome Analyses Reveal the Disruption of Lipid Metabolism in Systemic Lupus Erythematosus. Front Immunol (2020) 11:1703. doi: 10.3389/fimmu.2020.01703 32849599 PMC7411142

[29] Bellocchi C Fernandez-Ochoa A Montanelli G Vigone B Santaniello A Quirantes-Pine R. Identification of a Shared Microbiomic and Metabolomic Profile in Systemic Autoimmune Diseases. J Clin Med (2019) 8(9):1291. doi: 10.3390/jcm8091291 PMC6780636 31450824

[30] Azzouz D Omarbekova A Heguy A Schwudke D Gisch N Rovin BH. Lupus Nephritis is Linked to Disease-Activity Associated Expansions and Immunity to a Gut Commensal. Ann Rheum Dis (2019) 78(7):947–56. doi: 10.1136/annrheumdis-2018-214856 PMC6585303 30782585

[31] Chen BD Jia XM Xu JY Zhao LD Ji JY Wu BX. An Autoimmunogenic and Proinflammatory Profile Defined by the Gut Microbiota of Patients With Untreated Systemic Lupus Erythematosus. Arthritis Rheumatol (2021) 73(2):232–43. doi: 10.1002/art.41511 33124780

[32] Shi L Zhang Z Yu AM Wang W Wei Z Akhter E. The SLE Transcriptome Exhibits Evidence of Chronic Endotoxin Exposure and has Widespread Dysregulation of non-Coding and Coding RNAs. PLos One (2014) 9(5):e93846. doi: 10.1371/journal.pone.0093846 24796678 PMC4010412

[33] Ogunrinde E Zhou Z Luo Z Alekseyenko A Li QZ Macedo D. A Link Between Plasma Microbial Translocation, Microbiome, and Autoantibody Development in First-Degree Relatives of Systemic Lupus Erythematosus Patients. Arthritis Rheumatol (2019) 71(11):1858–68. doi: 10.1002/art.40935 PMC6817371 31106972

[34] Issara-Amphorn J Surawut S Worasilchai N Thim-Uam A Finkelman M Chindamporn A. The Synergy of Endotoxin and (1–>3)-Beta-D-Glucan, From Gut Translocation, Worsens Sepsis Severity in a Lupus Model of Fc Gamma Receptor IIb-Deficient Mice. J Innate Immun (2018) 10(3):189–201. doi: 10.1159/000486321 29393221 PMC6757155

[35] Manfredo Vieira S Hiltensperger M Kumar V Zegarra-Ruiz D Dehner C Khan N. Translocation of a Gut Pathobiont Drives Autoimmunity in Mice and Humans. Science (2018) 359(6380):1156–61. doi: 10.1126/science.aar7201 PMC5959731 29590047

[36] Nockher WA Wigand R Schoeppe W Scherberich JE. Elevated Levels of Soluble CD14 in Serum of Patients With Systemic Lupus Erythematosus. Clin Exp Immunol (1994) 96(1):15–9. doi: 10.1111/j.1365-2249.1994.tb06222.x PMC1534537 7512005

[37] Mu Q Tavella VJ Kirby JL Cecere TE Chung M Lee J. Antibiotics Ameliorate Lupus-Like Symptoms in Mice. Sci Rep (2017) 7(1):13675. doi: 10.1038/s41598-017-14223-0 29057975 PMC5651817

[38] Toral M Robles-Vera I Romero M de la Visitacion N Sanchez M O'Valle F. Lactobacillus Fermentum CECT5716: A Novel Alternative for the Prevention of Vascular Disorders in a Mouse Model of Systemic Lupus Erythematosus. FASEB J (2019) 33(9):10005–18. doi: 10.1096/fj.201900545RR 31173526

[39] Camilleri M. Leaky Gut: Mechanisms, Measurement and Clinical Implications in Humans. Gut (2019) 68(8):1516–26. doi: 10.1136/gutjnl-2019-318427 PMC6790068 31076401

[40] Thevaranjan N Puchta A Schulz C Naidoo A Szamosi JC Verschoor CP. Age-Associated Microbial Dysbiosis Promotes Intestinal Permeability, Systemic Inflammation, and Macrophage Dysfunction. Cell Host Microbe (2017) 21(4):455–66.e4. doi: 10.1016/j.chom.2017.03.002 28407483 PMC5392495

[41] Cani PD Delzenne NM. Interplay Between Obesity and Associated Metabolic Disorders: New Insights Into the Gut Microbiota. Curr Opin Pharmacol (2009) 9(6):737–43. doi: 10.1016/j.coph.2009.06.016 19628432

[42] Cario E Gerken G Podolsky DK. Toll-Like Receptor 2 Enhances ZO-1-Associated Intestinal Epithelial Barrier Integrity *via* Protein Kinase C. Gastroenterology (2004) 127(1):224–38. doi: 10.1053/j.gastro.2004.04.015 15236188

[43] Wang S Ahmadi S Nagpal R Jain S Mishra SP Kavanagh K. Lipoteichoic Acid From the Cell Wall of a Heat Killed Lactobacillus Paracasei D3-5 Ameliorates Aging-Related Leaky Gut, Inflammation and Improves Physical and Cognitive Functions: From C. Elegans to Mice. Geroscience (2020) 42(1):333–52. doi: 10.1007/s11357-019-00137-4 PMC7031475 31814084

[44] Karczewski J Troost FJ Konings I Dekker J Kleerebezem M Brummer RJ. Regulation of Human Epithelial Tight Junction Proteins by Lactobacillus Plantarum In Vivo and Protective Effects on the Epithelial Barrier. Am J Physiol Gastrointest Liver Physiol (2010) 298(6):G851–9. doi: 10.1152/ajpgi.00327.2009 20224007

[45] Xu J Liang R Zhang W Tian K Li J Chen X. Faecalibacterium Prausnitzii-Derived Microbial Anti-Inflammatory Molecule Regulates Intestinal Integrity in Diabetes Mellitus Mice *via* Modulating Tight Junction Protein Expression. J Diabetes (2020) 12(3):224–36. doi: 10.1111/1753-0407.12986 PMC7064962 31503404

[46] Chelakkot C Choi Y Kim DK Park HT Ghim J Kwon Y. Akkermansia Muciniphila-Derived Extracellular Vesicles Influence Gut Permeability Through the Regulation of Tight Junctions. Exp Mol Med (2018) 50(2):e450. doi: 10.1038/emm.2017.282 29472701 PMC5903829

[47] Miyauchi E O'Callaghan J Butto LF Hurley G Melgar S Tanabe S. Mechanism of Protection of Transepithelial Barrier Function by Lactobacillus Salivarius: Strain Dependence and Attenuation by Bacteriocin Production. Am J Physiol Gastrointest Liver Physiol (2012) 303(9):G1029–41. doi: 10.1152/ajpgi.00003.2012 22961803

[48] Sofi MH Wu Y Ticer T Schutt S Bastian D Choi HJ. A Single Strain of Bacteroides Fragilis Protects Gut Integrity and Reduces GVHD. JCI Insight (2021) 6(3):e136841. doi: 10.1172/jci.insight.136841 PMC7934839 33554953

[49] Zhang H Liu M Zhong W Zheng Y Li Y Guo L. Leaky Gut Driven by Dysbiosis Augments Activation and Accumulation of Liver Macrophages *via* RIP3 Signaling Pathway in Autoimmune Hepatitis. Front Immunol (2021) 12:624360. doi: 10.3389/fimmu.2021.624360 33841405 PMC8027109

[50] Yu LC. Microbiota Dysbiosis and Barrier Dysfunction in Inflammatory Bowel Disease and Colorectal Cancers: Exploring a Common Ground Hypothesis. J BioMed Sci (2018) 25(1):79. doi: 10.1186/s12929-018-0483-8 30413188 PMC6234774

[51] Wick EC Rabizadeh S Albesiano E Wu X Wu S Chan J. Stat3 Activation in Murine Colitis Induced by Enterotoxigenic Bacteroides Fragilis. Inflamm Bowel Dis (2014) 20(5):821–34. doi: 10.1097/MIB.0000000000000019 PMC4121853 24704822

[52] Obiso RJ Jr. Azghani AO Wilkins TD. The Bacteroides Fragilis Toxin Fragilysin Disrupts the Paracellular Barrier of Epithelial Cells. Infect Immun (1997) 65(4):1431–9. doi: 10.1128/iai.65.4.1431-1439.1997 PMC175150 9119484

[53] Hecht G Pothoulakis C LaMont JT Madara JL. Clostridium Difficile Toxin A Perturbs Cytoskeletal Structure and Tight Junction Permeability of Cultured Human Intestinal Epithelial Monolayers. J Clin Invest (1988) 82(5):1516–24. doi: 10.1172/JCI113760 PMC442717 3141478

[54] Eichner M Protze J Piontek A Krause G Piontek J. Targeting and Alteration of Tight Junctions by Bacteria and Their Virulence Factors Such as Clostridium Perfringens Enterotoxin. Pflugers Arch (2017) 469(1):77–90. doi: 10.1007/s00424-016-1902-x 27864644

[55] Mirsepasi-Lauridsen HC Du Z Struve C Charbon G Karczewski J Krogfelt KA. Secretion of Alpha-Hemolysin by Escherichia Coli Disrupts Tight Junctions in Ulcerative Colitis Patients. Clin Transl Gastroenterol (2016) 7:e149. doi: 10.1038/ctg.2016.3 26938480 PMC4822097

[56] Papini E Satin B Norais N de Bernard M Telford JL Rappuoli R. Selective Increase of the Permeability of Polarized Epithelial Cell Monolayers by Helicobacter Pylori Vacuolating Toxin. J Clin Invest (1998) 102(4):813–20. doi: 10.1172/JCI2764 PMC508944 9710450

[57] Mathipa MG Bhunia AK Thantsha MS. Internalin AB-Expressing Recombinant Lactobacillus Casei Protects Caco-2 Cells From Listeria Monocytogenes-Induced Damages Under Simulated Intestinal Conditions. PLos One (2019) 14(7):e0220321. doi: 10.1371/journal.pone.0220321 31356632 PMC6663025

[58] Fasano A Fiorentini C Donelli G Uzzau S Kaper JB Margaretten K. Zonula Occludens Toxin Modulates Tight Junctions Through Protein Kinase C-Dependent Actin Reorganization, In Vitro. J Clin Invest (1995) 96(2):710–20. doi: 10.1172/JCI118114 PMC185254 7635964

[59] Li C Gao M Zhang W Chen C Zhou F Hu Z. Zonulin Regulates Intestinal Permeability and Facilitates Enteric Bacteria Permeation in Coronary Artery Disease. Sci Rep (2016) 6:29142. doi: 10.1038/srep29142 27353603 PMC4926221

[60] Dagci H Ustun S Taner MS Ersoz G Karacasu F Budak S. Protozoon Infections and Intestinal Permeability. Acta Trop (2002) 81(1):1–5. doi: 10.1016/S0001-706X(01)00191-7 11755426

[61] Beau I Cotte-Laffitte J Amsellem R Servin AL. A Protein Kinase A-Dependent Mechanism by Which Rotavirus Affects the Distribution and mRNA Level of the Functional Tight Junction-Associated Protein, Occludin, in Human Differentiated Intestinal Caco-2 Cells. J Virol (2007) 81(16):8579–86. doi: 10.1128/JVI.00263-07 PMC1951370 17553883

[62] Kulkarni OP Lichtnekert J Anders HJ Mulay SR. The Immune System in Tissue Environments Regaining Homeostasis After Injury: Is "Inflammation" Always Inflammation? Mediators Inflamm (2016) 2016:2856213. doi: 10.1155/2016/2856213 27597803 PMC4997018

[63] Zeng MY Inohara N Nunez G. Mechanisms of Inflammation-Driven Bacterial Dysbiosis in the Gut. Mucosal Immunol (2017) 10(1):18–26. doi: 10.1038/mi.2016.75 27554295 PMC5788567

[64] Thim-Uam A Surawut S Issara-Amphorn J Jaroonwitchawan T Hiengrach P Chatthanathon P. Leaky-Gut Enhanced Lupus Progression in the Fc Gamma Receptor-IIb Deficient and Pristane-Induced Mouse Models of Lupus. Sci Rep (2020) 10(1):777. doi: 10.1038/s41598-019-57275-0 31964918 PMC6972921

[65] Sorini C Cosorich I Lo Conte M De Giorgi L Facciotti F Luciano R. Loss of Gut Barrier Integrity Triggers Activation of Islet-Reactive T Cells and Autoimmune Diabetes. Proc Natl Acad Sci U S A (2019) 116(30):15140–9. doi: 10.1073/pnas.1814558116 PMC6660755 31182588

[66] McCole DF. IBD Candidate Genes and Intestinal Barrier Regulation. Inflamm Bowel Dis (2014) 20(10):1829–49. doi: 10.1097/MIB.0000000000000090 PMC4357271 25215613

[67] Xavier RJ Podolsky DK. Unravelling the Pathogenesis of Inflammatory Bowel Disease. Nature (2007) 448(7152):427–34. doi: 10.1038/nature06005 17653185

[68] Buhner S Buning C Genschel J Kling K Herrmann D Dignass A. Genetic Basis for Increased Intestinal Permeability in Families With Crohn's Disease: Role of CARD15 3020insc Mutation? Gut (2006) 55(3):342–7. doi: 10.1136/gut.2005.065557 PMC1856071 16000642

[69] Clemente MG De Virgiliis S Kang JS Macatagney R Musu MP Di Pierro MR. Early Effects of Gliadin on Enterocyte Intracellular Signalling Involved in Intestinal Barrier Function. Gut (2003) 52(2):218–23. doi: 10.1136/gut.52.2.218 PMC1774976 12524403

[70] Elli L Roncoroni L Doneda L Ciulla MM Colombo R Braidotti P. Imaging Analysis of the Gliadin Direct Effect on Tight Junctions in an *In Vitro* Three-Dimensional Lovo Cell Line Culture System. Toxicol In Vitro (2011) 25(1):45–50. doi: 10.1016/j.tiv.2010.09.005 20850517

[71] Sander GR Cummins AG Henshall T Powell BC. Rapid Disruption of Intestinal Barrier Function by Gliadin Involves Altered Expression of Apical Junctional Proteins. FEBS Lett (2005) 579(21):4851–5. doi: 10.1016/j.febslet.2005.07.066 16099460

[72] Tollefsen S Arentz-Hansen H Fleckenstein B Molberg O Raki M Kwok WW. HLA-DQ2 and -DQ8 Signatures of Gluten T Cell Epitopes in Celiac Disease. J Clin Invest (2006) 116(8):2226–36. doi: 10.1172/JCI27620 PMC1518792 16878175

[73] Langefeld CD Ainsworth HC Cunninghame Graham DS Kelly JA Comeau ME Marion MC. Transancestral Mapping and Genetic Load in Systemic Lupus Erythematosus. Nat Commun (2017) 8:16021. doi: 10.1038/ncomms16021 28714469 PMC5520018

[74] Janeckova L Kostovcikova K Svec J Stastna M Strnad H Kolar M. Unique Gene Expression Signatures in the Intestinal Mucosa and Organoids Derived From Germ-Free and Monoassociated Mice. Int J Mol Sci (2019) 20(7):1581. doi: 10.3390/ijms20071581 PMC6480644 30934845

[75] Pacheco GV Nakazawa Ueji YE Bello JR Barbosa Cobos RE Jimenez Becerra ED Gonzalez Herrera LJ. Copy Number Variation and Frequency of Rs179008 in TLR7 Gene Associated With Systemic Lupus Erythematosus in Two Mexican Populations. J Immunol Res (2022) 2022:2553901. doi: 10.1155/2022/2553901 35083340 PMC8786460

[76] Perl A Fernandez DR Telarico T Doherty E Francis L Phillips PE. T-Cell and B-Cell Signaling Biomarkers and Treatment Targets in Lupus. Curr Opin Rheumatol (2009) 21(5):454–64. doi: 10.1097/BOR.0b013e32832e977c PMC4047522 19550330

[77] Hepburn AL Mason JC Davies KA. Expression of Fcgamma and Complement Receptors on Peripheral Blood Monocytes in Systemic Lupus Erythematosus and Rheumatoid Arthritis. Rheumatol (Ox) (2004) 43(5):547–54. doi: 10.1093/rheumatology/keh112 14747618

[78] Li Y Lee PY Reeves WH. Monocyte and Macrophage Abnormalities in Systemic Lupus Erythematosus. Arch Immunol Ther Exp (Warsz) (2010) 58(5):355–64. doi: 10.1007/s00005-010-0093-y PMC3785254 20676786

[79] Cliffe LJ Humphreys NE Lane TE Potten CS Booth C Grencis RK. Accelerated Intestinal Epithelial Cell Turnover: A New Mechanism of Parasite Expulsion. Science (2005) 308(5727):1463–5. doi: 10.1126/science.1108661 15933199

[80] Ghorbaninezhad F Leone P Alemohammad H Najafzadeh B Nourbakhsh NS Prete M. Tumor Necrosis Factoralpha in Systemic Lupus Erythematosus: Structure, Function and Therapeutic Implications (Review). Int J Mol Med (2022) 49(4):1–13. doi: 10.3892/ijmm.2022.5098 35137914

[81] Shin JI Lee KH Joo YH Lee JM Jeon J Jung HJ. Inflammasomes and Autoimmune and Rheumatic Diseases: A Comprehensive Review. J Autoimmun (2019) 103:102299. doi: 10.1016/j.jaut.2019.06.010 31326231

[82] Oke V Gunnarsson I Dorschner J Eketjall S Zickert A Niewold TB. And Type III Associate With Distinct Clinical Features of Active Systemic Lupus Erythematosus. Arthritis Res Ther (2019) 21(1):107. doi: 10.1186/s13075-019-1878-y 31036046 PMC6489203

[83] Al-Sadi R Boivin M Ma T. Mechanism of Cytokine Modulation of Epithelial Tight Junction Barrier. Front Biosci (Landmark Ed) (2009) 14(7):2765–78. doi: 10.2741/3413 PMC3724223 19273235

[84] Noval Rivas M Wakita D Franklin MK Carvalho TT Abolhesn A Gomez AC. Intestinal Permeability and IgA Provoke Immune Vasculitis Linked to Cardiovascular Inflammation. Immunity (2019) 51(3):508–21.e6. doi: 10.1016/j.immuni.2019.05.021 31471109 PMC6751009

[85] Ma TY Iwamoto GK Hoa NT Akotia V Pedram A Boivin MA. TNF-Alpha-Induced Increase in Intestinal Epithelial Tight Junction Permeability Requires NF-Kappa B Activation. Am J Physiol Gastrointest Liver Physiol (2004) 286(3):G367–76. doi: 10.1152/ajpgi.00173.2003 14766535

[86] Al-Sadi RM Ma TY. IL-1beta Causes an Increase in Intestinal Epithelial Tight Junction Permeability. J Immunol (2007) 178(7):4641–9. doi: 10.4049/jimmunol.178.7.4641 PMC3724221 17372023

[87] Malynn BA Ma A. A20: A Multifunctional Tool for Regulating Immunity and Preventing Disease. Cell Immunol (2019) 340:103914. doi: 10.1016/j.cellimm.2019.04.002 31030956 PMC6584049

[88] Nenci A Becker C Wullaert A Gareus R van Loo G Danese S. Epithelial NEMO Links Innate Immunity to Chronic Intestinal Inflammation. Nature (2007) 446(7135):557–61. doi: 10.1038/nature05698 17361131

[89] Battaglia M Garrett-Sinha LA. Bacterial Infections in Lupus: Roles in Promoting Immune Activation and in Pathogenesis of the Disease. J Transl Autoimmun (2021) 4:100078. doi: 10.1016/j.jtauto.2020.100078 33490939 PMC7804979

[90] Zhang YG Wu S Xia Y Sun J. Salmonella Infection Upregulates the Leaky Protein Claudin-2 in Intestinal Epithelial Cells. PLos One (2013) 8(3):e58606. doi: 10.1371/journal.pone.0058606 23505542 PMC3594366

[91] Nakajima M Arimatsu K Kato T Matsuda Y Minagawa T Takahashi N. Oral Administration of P. Gingivalis Induces Dysbiosis of Gut Microbiota and Impaired Barrier Function Leading to Dissemination of Enterobacteria to the Liver. PLos One (2015) 10(7):e0134234. doi: 10.1371/journal.pone.0134234 26218067 PMC4517782

[92] Liu H Hong XL Sun TT Huang XW Wang JL Xiong H. Fusobacterium Nucleatum Exacerbates Colitis by Damaging Epithelial Barriers and Inducing Aberrant Inflammation. J Dig Dis (2020) 21(7):385–98. doi: 10.1111/1751-2980.12909 32441482

[93] Routy JP Royston L Isnard S. Aging With Grace for People Living With HIV: Strategies to Overcome Leaky Gut and Cytomegalovirus Coinfection. J Acquir Immune Defic Syndr (2022) 89(Suppl 1):S29–33. doi: 10.1097/QAI.0000000000002838 PMC8751289 35015743

[94] Tetz G Tetz V. Bacteriophage Infections of Microbiota can Lead to Leaky Gut in an Experimental Rodent Model. Gut Pathog (2016) 8:33. doi: 10.1186/s13099-016-0109-1 27340433 PMC4918031

[95] Chancharoenthana W Leelahavanichkul A Ariyanon W Vadcharavivad S Phatcharophaswattanakul S Kamolratanakul S. Leaky Gut Syndrome Is Associated With Endotoxemia and Serum (1–>3)-Beta-D-Glucan in Severe Dengue Infection. Microorganisms (2021) 9(11):2390. doi: 10.3390/microorganisms9112390 34835514 PMC8625530

[96] Asarat M Apostolopoulos V Vasiljevic T Donkor O. Short-Chain Fatty Acids Regulate Cytokines and Th17/Treg Cells in Human Peripheral Blood Mononuclear Cells In Vitro. Immunol Invest (2016) 45(3):205–22. doi: 10.3109/08820139.2015.1122613 27018846

[97] Fukuda S Toh H Hase K Oshima K Nakanishi Y Yoshimura K. Bifidobacteria can Protect From Enteropathogenic Infection Through Production of Acetate. Nature (2011) 469(7331):543–7. doi: 10.1038/nature09646 21270894

[98] Kelly CJ Zheng L Campbell EL Saeedi B Scholz CC Bayless AJ. Crosstalk Between Microbiota-Derived Short-Chain Fatty Acids and Intestinal Epithelial HIF Augments Tissue Barrier Function. Cell Host Microbe (2015) 17(5):662–71. doi: 10.1016/j.chom.2015.03.005 PMC4433427 25865369

[99] Peng L He Z Chen W Holzman IR Lin J. Effects of Butyrate on Intestinal Barrier Function in a Caco-2 Cell Monolayer Model of Intestinal Barrier. Pediatr Res (2007) 61(1):37–41. doi: 10.1203/01.pdr.0000250014.92242.f3 17211138

[100] Peng L Li ZR Green RS Holzman IR Lin J. Butyrate Enhances the Intestinal Barrier by Facilitating Tight Junction Assembly *via* Activation of AMP-Activated Protein Kinase in Caco-2 Cell Monolayers. J Nutr (2009) 139(9):1619–25. doi: 10.3945/jn.109.104638 PMC2728689 19625695

[101] Hsieh CY Osaka T Moriyama E Date Y Kikuchi J Tsuneda S. Strengthening of the Intestinal Epithelial Tight Junction by Bifidobacterium Bifidum. Physiol Rep (2015) 3(3):e12327. doi: 10.14814/phy2.12327 25780093 PMC4393161

[102] Brown J Robusto B Morel L. Intestinal Dysbiosis and Tryptophan Metabolism in Autoimmunity. Front Immunol (2020) 11:1741. doi: 10.3389/fimmu.2020.01741 32849620 PMC7417361

[103] Bansal T Alaniz RC Wood TK Jayaraman A. The Bacterial Signal Indole Increases Epithelial-Cell Tight-Junction Resistance and Attenuates Indicators of Inflammation. Proc Natl Acad Sci USA (2010) 107(1):228–33. doi: 10.1073/pnas.0906112107 PMC2806735 19966295

[104] Shimada Y Kinoshita M Harada K Mizutani M Masahata K Kayama H. Commensal Bacteria-Dependent Indole Production Enhances Epithelial Barrier Function in the Colon. PLos One (2013) 8(11):e80604. doi: 10.1371/journal.pone.0080604 24278294 PMC3835565

[105] Le Bastard Q Al-Ghalith GA Gregoire M Chapelet G Javaudin F Dailly E. Systematic Review: Human Gut Dysbiosis Induced by non-Antibiotic Prescription Medications. Aliment Pharmacol Ther (2018) 47(3):332–45. doi: 10.1111/apt.14451 29205415

[106] Duan H Yu L Tian F Zhai Q Fan L Chen W. Antibiotic-Induced Gut Dysbiosis and Barrier Disruption and the Potential Protective Strategies. Crit Rev Food Sci Nutr (2022) 62(6):1427–52. doi: 10.1080/10408398.2020.1843396 33198506

[107] Singh G Ramey DR Morfeld D Shi H Hatoum HT Fries JF. Gastrointestinal Tract Complications of Nonsteroidal Anti-Inflammatory Drug Treatment in Rheumatoid Arthritis. A Prospective Observational Cohort Study. Arch Intern Med (1996) 156(14):1530–6. doi: 10.1001/archinte.1996.00440130066007 8687261

[108] Braga CB Martins AC Cayotopa AD Klein WW Schlosser AR da Silva AF. Side Effects of Chloroquine and Primaquine and Symptom Reduction in Malaria Endemic Area (Mancio Lima, Acre, Brazil). Interdiscip Perspect Infect Dis (2015) 2015:346853. doi: 10.1155/2015/346853 26357512 PMC4556080

[109] Mullins JF Watts FL Wilson CJ. Plaquenil in the Treatment of Lupus Erythematosus. J Am Med Assoc (1956) 161(9):879–81. doi: 10.1001/jama.1956.62970090020017k 13319032

[110] Bjarnason I Scarpignato C Holmgren E Olszewski M Rainsford KD Lanas A. Mechanisms of Damage to the Gastrointestinal Tract From Nonsteroidal Anti-Inflammatory Drugs. Gastroenterology (2018) 154(3):500–14. doi: 10.1053/j.gastro.2017.10.049 29221664

[111] Madsen KL Yanchar NL Sigalet DL Reigel T Fedorak RN. FK506 Increases Permeability in Rat Intestine by Inhibiting Mitochondrial Function. Gastroenterology (1995) 109(1):107–14. doi: 10.1016/0016-5085(95)90274-0 7540994

[112] Clarke J. Voclosporin Improves Outcomes in Lupus Nephritis. Nat Rev Rheumatol (2021) 17(7):378. doi: 10.1038/s41584-021-00638-7 34040229

[113] Danza A Ruiz-Irastorza G. Infection Risk in Systemic Lupus Erythematosus Patients: Susceptibility Factors and Preventive Strategies. Lupus (2013) 22(12):1286–94. doi: 10.1177/0961203313493032 24098001

[114] Kang I Park SH. Infectious Complications in SLE After Immunosuppressive Therapies. Curr Opin Rheumatol (2003) 15(5):528–34. doi: 10.1097/00002281-200309000-00002 12960476

[115] Doaty S Agrawal H Bauer E Furst DE. Infection and Lupus: Which Causes Which? Curr Rheumatol Rep (2016) 18(3):13. doi: 10.1007/s11926-016-0561-4 26951251

[116] Takeuchi O Akira S. Pattern Recognition Receptors and Inflammation. Cell (2010) 140(6):805–20. doi: 10.1016/j.cell.2010.01.022 20303872

[117] Gotoh M Matsuda J. Induction of Anticardiolipin Antibody and/or Lupus Anticoagulant in Rabbits by Immunization With Lipoteichoic Acid, Lipopolysaccharide and Lipid a. Lupus (1996) 5(6):593–7. doi: 10.1177/096120339600500606 9116702

[118] Ginsburg I. Role of Lipoteichoic Acid in Infection and Inflammation. Lancet Infect Dis (2002) 2(3):171–9. doi: 10.1016/S1473-3099(02)00226-8 11944187

[119] Gallo PM Rapsinski GJ Wilson RP Oppong GO Sriram U Goulian M. Amyloid-DNA Composites of Bacterial Biofilms Stimulate Autoimmunity. Immunity (2015) 42(6):1171–84. doi: 10.1016/j.immuni.2015.06.002 PMC4500125 26084027

[120] Guerreiro CS Calado A Sousa J Fonseca JE. Diet, Microbiota, and Gut Permeability-The Unknown Triad in Rheumatoid Arthritis. Front Med (Lausanne) (2018) 5:349. doi: 10.3389/fmed.2018.00349 30619860 PMC6302746

[121] Cani PD Bibiloni R Knauf C Waget A Neyrinck AM Delzenne NM. Changes in Gut Microbiota Control Metabolic Endotoxemia-Induced Inflammation in High-Fat Diet-Induced Obesity and Diabetes in Mice. Diabetes (2008) 57(6):1470–81. doi: 10.2337/db07-1403 18305141

[122] Kirpich IA Feng W Wang Y Liu Y Barker DF Barve SS. The Type of Dietary Fat Modulates Intestinal Tight Junction Integrity, Gut Permeability, and Hepatic Toll-Like Receptor Expression in a Mouse Model of Alcoholic Liver Disease. Alcohol Clin Exp Res (2012) 36(5):835–46. doi: 10.1111/j.1530-0277.2011.01673.x PMC3319492 22150547

[123] Suzuki T Hara H. Dietary Fat and Bile Juice, But Not Obesity, are Responsible for the Increase in Small Intestinal Permeability Induced Through the Suppression of Tight Junction Protein Expression in LETO and OLETF Rats. Nutr Metab (Lond) (2010) 7:19. doi: 10.1186/1743-7075-7-19 20222989 PMC2848226

[124] Araki Y Katoh T Ogawa A Bamba S Andoh A Koyama S. Bile Acid Modulates Transepithelial Permeability *via* the Generation of Reactive Oxygen Species in the Caco-2 Cell Line. Free Radic Biol Med (2005) 39(6):769–80. doi: 10.1016/j.freeradbiomed.2005.04.026 16109307

[125] Raimondi F Santoro P Barone MV Pappacoda S Barretta ML Nanayakkara M. Bile Acids Modulate Tight Junction Structure and Barrier Function of Caco-2 Monolayers *via* EGFR Activation. Am J Physiol Gastrointest Liver Physiol (2008) 294(4):G906–13. doi: 10.1152/ajpgi.00043.2007 18239063

[126] Desai MS Seekatz AM Koropatkin NM Kamada N Hickey CA Wolter M. A Dietary Fiber-Deprived Gut Microbiota Degrades the Colonic Mucus Barrier and Enhances Pathogen Susceptibility. Cell (2016) 167(5):1339–53.e21. doi: 10.1016/j.cell.2016.10.043 27863247 PMC5131798

[127] Khan S Gaivin R Abramovich C Boylan M Calles J Schelling JR. Fatty Acid Transport Protein-2 Regulates Glycemic Control and Diabetic Kidney Disease Progression. JCI Insight (2020) 5(15):e136845. doi: 10.1172/jci.insight.136845 PMC7455077 32614804

[128] Zhang C Zhang M Pang X Zhao Y Wang L Zhao L. Structural Resilience of the Gut Microbiota in Adult Mice Under High-Fat Dietary Perturbations. ISME J (2012) 6(10):1848–57. doi: 10.1038/ismej.2012.27 PMC3446802 22495068

[129] Hanna Kazazian N Wang Y Roussel-Queval A Marcadet L Chasson L Laprie C. Lupus Autoimmunity and Metabolic Parameters Are Exacerbated Upon High Fat Diet-Induced Obesity Due to TLR7 Signaling. Front Immunol (2019) 10:2015. doi: 10.3389/fimmu.2019.02015 31552019 PMC6738575

[130] Kostovcikova K Coufal S Galanova N Fajstova A Hudcovic T Kostovcik M. Diet Rich in Animal Protein Promotes Pro-Inflammatory Macrophage Response and Exacerbates Colitis in Mice. Front Immunol (2019) 10:919. doi: 10.3389/fimmu.2019.00919 31105710 PMC6497971

[131] Do MH Lee E Oh MJ Kim Y Park HY. High-Glucose or -Fructose Diet Cause Changes of the Gut Microbiota and Metabolic Disorders in Mice Without Body Weight Change. Nutrients (2018) 10(6):761. doi: 10.3390/nu10060761 PMC6024874 29899272

[132] Chung CP Avalos I Oeser A Gebretsadik T Shintani A Raggi P. High Prevalence of the Metabolic Syndrome in Patients With Systemic Lupus Erythematosus: Association With Disease Characteristics and Cardiovascular Risk Factors. Ann Rheum Dis (2007) 66(2):208–14. doi: 10.1136/ard.2006.054973 PMC1798504 16901956

[133] Chassaing B Vijay-Kumar M Gewirtz AT. How Diet can Impact Gut Microbiota to Promote or Endanger Health. Curr Opin Gastroenterol (2017) 33(6):417–21. doi: 10.1097/MOG.0000000000000401 PMC6005665 29019865

[134] Cutolo M Otsa K. Review: Vitamin D, Immunity and Lupus. Lupus (2008) 17(1):6–10. doi: 10.1177/0961203307085879 18089676

[135] Ruiz-Irastorza G Egurbide MV Olivares N Martinez-Berriotxoa A Aguirre C. Vitamin D Deficiency in Systemic Lupus Erythematosus: Prevalence, Predictors and Clinical Consequences. Rheumatol (Oxf) (2008) 47(6):920–3. doi: 10.1093/rheumatology/ken121 18411213

[136] Yap KS Northcott M Hoi AB Morand EF Nikpour M. Association of Low Vitamin D With High Disease Activity in an Australian Systemic Lupus Erythematosus Cohort. Lupus Sci Med (2015) 2(1):e000064. doi: 10.1136/lupus-2014-000064 25893106 PMC4395813

[137] Abdelhamid L Luo XM. Retinoic Acid, Leaky Gut, and Autoimmune Diseases. Nutrients (2018) 10(8):1016. doi: 10.3390/nu10081016 PMC6115935 30081517

[138] Lima AA Brito LF Ribeiro HB Martins MC Lustosa AP Rocha EM. Intestinal Barrier Function and Weight Gain in Malnourished Children Taking Glutamine Supplemented Enteral Formula. J Pediatr Gastroenterol Nutr (2005) 40(1):28–35. doi: 10.1097/00005176-200501000-00006 15625423

[139] Ouyang X Dai Y Wen JL Wang LX. (1)H NMR-Based Metabolomic Study of Metabolic Profiling for Systemic Lupus Erythematosus. Lupus (2011) 20(13):1411–20. doi: 10.1177/0961203311418707 21976403

[140] Yan B Huang J Zhang C Hu X Gao M Shi A. Serum Metabolomic Profiling in Patients With Systemic Lupus Erythematosus by GC/MS. Mod Rheumatol (2016) 26(6):914–22. doi: 10.3109/14397595.2016.1158895 26915395

[141] Vanuytsel T van Wanrooy S Vanheel H Vanormelingen C Verschueren S Houben E. Psychological Stress and Corticotropin-Releasing Hormone Increase Intestinal Permeability in Humans by a Mast Cell-Dependent Mechanism. Gut (2014) 63(8):1293–9. doi: 10.1136/gutjnl-2013-305690 24153250

[142] Park AJ Collins J Blennerhassett PA Ghia JE Verdu EF Bercik P. Altered Colonic Function and Microbiota Profile in a Mouse Model of Chronic Depression. Neurogastroenterol Motil (2013) 25(9):733–e575. doi: 10.1111/nmo.12153 23773726 PMC3912902

[143] Madison A Kiecolt-Glaser JK. Stress, Depression, Diet, and the Gut Microbiota: Human-Bacteria Interactions at the Core of Psychoneuroimmunology and Nutrition. Curr Opin Behav Sci (2019) 28:105–10. doi: 10.1016/j.cobeha.2019.01.011 PMC7213601 32395568

[144] Olesinska M Saletra A. Quality of Life in Systemic Lupus Erythematosus and its Measurement. Reumatologia (2018) 56(1):45–54. doi: 10.5114/reum.2018.74750 29686443 PMC5911658

[145] Gopalakrishnan S Durai M Kitchens K Tamiz AP Somerville R Ginski M. Larazotide Acetate Regulates Epithelial Tight Junctions *In Vitro* and In Vivo. Peptides (2012) 35(1):86–94. doi: 10.1016/j.peptides.2012.02.015 22401908

[146] Slifer ZM Krishnan BR Madan J Blikslager AT. Larazotide Acetate: A Pharmacological Peptide Approach to Tight Junction Regulation. Am J Physiol Gastrointest Liver Physiol (2021) 320(6):G983–G9. doi: 10.1152/ajpgi.00386.2020 PMC11735010 33881350

[147] Rittirsch D Flierl MA Nadeau BA Day DE Huber-Lang MS Grailer JJ. Zonulin as Prehaptoglobin2 Regulates Lung Permeability and Activates the Complement System. Am J Physiol Lung Cell Mol Physiol (2013) 304(12):L863–72. doi: 10.1152/ajplung.00196.2012 PMC3680747 23564505

[148] Fasano A. Leaky Gut and Autoimmune Diseases. Clin Rev Allergy Immunol (2012) 42(1):71–8. doi: 10.1007/s12016-011-8291-x 22109896

[149] Gudi R Kamen D Vasu C. Differences in the Gut Permeability Marker Levels, and Abundance and Nuclear Antigen Reactivity of Fecal Immunoglobulin A (IgA) Subclasses in Systemic Lupus Erythematosus Patients and Healthy Controls. bioRxiv (2022). doi: 10.1101/2022.01.26.477918 PMC10785702 36049603

[150] Deng J Azzouz DF Ferstler N Silverman GJ. Sex-Dependent Lupus Ruminococcus Blautia Gnavus Strain Induction of Zonulin-Mediated Intestinal Permeability and Autoimmunity. bioRxiv (2021). doi: 10.1101/2021.07.06.451365 PMC9405438 36032126

[151] Elbere I Kalnina I Silamikelis I Konrade I Zaharenko L Sekace K. Association of Metformin Administration With Gut Microbiome Dysbiosis in Healthy Volunteers. PLos One (2018) 13(9):e0204317. doi: 10.1371/journal.pone.0204317 30261008 PMC6160085

[152] Liang H Song H Zhang X Song G Wang Y Ding X. Metformin Attenuated Sepsis-Related Liver Injury by Modulating Gut Microbiota. Emerg Microbes Infect (2022) 11(1):815–28. doi: 10.1080/22221751.2022.2045876 PMC8928825 35191819

[153] Ahmadi S Razazan A Nagpal R Jain S Wang B Mishra SP. Metformin Reduces Aging-Related Leaky Gut and Improves Cognitive Function by Beneficially Modulating Gut Microbiome/Goblet Cell/Mucin Axis. J Gerontol A Biol Sci Med Sci (2020) 75(7):e9–e21. doi: 10.1093/gerona/glaa056 32129462 PMC7302182

[154] Silamikele L Silamikelis I Ustinova M Kalnina Z Elbere I Petrovska R. Metformin Strongly Affects Gut Microbiome Composition in High-Fat Diet-Induced Type 2 Diabetes Mouse Model of Both Sexes. Front Endocrinol (Lausanne) (2021) 12:626359. doi: 10.3389/fendo.2021.626359 33815284 PMC8018580

[155] Yin Y Choi SC Xu Z Perry DJ Seay H Croker BP. Normalization of CD4+ T Cell Metabolism Reverses Lupus. Sci Transl Med (2015) 7(274):274ra18. doi: 10.1126/scitranslmed.aaa0835 PMC5292723 25673763

[156] Sun F Geng S Wang H Wang H Liu Z Wang X. Effects of Metformin on Disease Flares in Patients With Systemic Lupus Erythematosus: Post Hoc Analyses From Two Randomised Trials. Lupus Sci Med (2020) 7(1):e000429. doi: 10.1136/lupus-2020-000429 33093216 PMC7583791

[157] Titov AA Baker HV Brusko TM Sobel ES Morel L. Metformin Inhibits the Type 1 IFN Response in Human CD4(+) T Cells. J Immunol (2019) 203(2):338–48. doi: 10.4049/jimmunol.1801651 PMC6615983 31160534

[158] Cao M Wang P Sun C He W Wang F. Amelioration of IFN-Gamma and TNF-Alpha-Induced Intestinal Epithelial Barrier Dysfunction by Berberine *via* Suppression of MLCK-MLC Phosphorylation Signaling Pathway. PLos One (2013) 8(5):e61944. doi: 10.1371/journal.pone.0061944 23671580 PMC3643960

[159] Gu L Li N Li Q Zhang Q Wang C Zhu W. The Effect of Berberine *In Vitro* on Tight Junctions in Human Caco-2 Intestinal Epithelial Cells. Fitoterapia (2009) 80(4):241–8. doi: 10.1016/j.fitote.2009.02.005 19243699

[160] Lu Q Fu Y Li H. Berberine and its Derivatives Represent as the Promising Therapeutic Agents for Inflammatory Disorders. Pharmacol Rep (2022) 74(2):297–309. doi: 10.1007/s43440-021-00348-7 35083737

[161] Yang T Aquino V Lobaton GO Li H Colon-Perez L Goel R. Sustained Captopril-Induced Reduction in Blood Pressure Is Associated With Alterations in Gut-Brain Axis in the Spontaneously Hypertensive Rat. J Am Heart Assoc (2019) 8(4):e010721. doi: 10.1161/JAHA.118.010721 30755073 PMC6405665

[162] Reiberger T Ferlitsch A Payer BA Mandorfer M Heinisch BB Hayden H. Non-Selective Betablocker Therapy Decreases Intestinal Permeability and Serum Levels of LBP and IL-6 in Patients With Cirrhosis. J Hepatol (2013) 58(5):911–21. doi: 10.1016/j.jhep.2012.12.011 23262249

[163] Kuti D Winkler Z Horvath K Juhasz B Paholcsek M Stagel A. Gastrointestinal (non-Systemic) Antibiotic Rifaximin Differentially Affects Chronic Stress-Induced Changes in Colon Microbiome and Gut Permeability Without Effect on Behavior. Brain Behav Immun (2020) 84:218–28. doi: 10.1016/j.bbi.2019.12.004 31821847

[164] Kaji K Saikawa S Takaya H Fujinaga Y Furukawa M Kitagawa K. Rifaximin Alleviates Endotoxemia With Decreased Serum Levels of Soluble CD163 and Mannose Receptor and Partial Modification of Gut Microbiota in Cirrhotic Patients. Antibiotics (Basel) (2020) 9(4):145. doi: 10.3390/antibiotics9040145 PMC7235723 32235367

[165] Lerang K Gilboe IM Steinar Thelle D Gran JT. Mortality and Years of Potential Life Loss in Systemic Lupus Erythematosus: A Population-Based Cohort Study. Lupus (2014) 23(14):1546–52. doi: 10.1177/0961203314551083 25209070

[166] Xu D Gao J Gillilland M 3rd Wu X Song I Kao JY. Rifaximin Alters Intestinal Bacteria and Prevents Stress-Induced Gut Inflammation and Visceral Hyperalgesia in Rats. Gastroenterology (2014) 146(2):484–96.e4. doi: 10.1053/j.gastro.2013.10.026 24161699 PMC3939606

[167] Ghosh SS Bie J Wang J Ghosh S. Oral Supplementation With non-Absorbable Antibiotics or Curcumin Attenuates Western Diet-Induced Atherosclerosis and Glucose Intolerance in LDLR-/- Mice–Role of Intestinal Permeability and Macrophage Activation. PLos One (2014) 9(9):e108577. doi: 10.1371/journal.pone.0108577 25251395 PMC4177397

[168] Hu J Luo H Wang J Tang W Lu J Wu S. Enteric Dysbiosis-Linked Gut Barrier Disruption Triggers Early Renal Injury Induced by Chronic High Salt Feeding in Mice. Exp Mol Med (2017) 49(8):e370. doi: 10.1038/emm.2017.122 28857085 PMC5579512

[169] James WA Ogunrinde E Wan Z Kamen DL Oates J Gilkeson GS. A Distinct Plasma Microbiome But Not Gut Microbiome in Patients With Systemic Lupus Erythematosus Compared to Healthy Individuals. J Rheumatol (2022) 49(6):592–97. doi: 10.3899/jrheum.210952 PMC9364828 35169056

[170] Plaza-Diaz J Ruiz-Ojeda FJ Gil-Campos M Gil A. Mechanisms of Action of Probiotics. Adv Nutr (2019) 10(suppl_1):S49–66. doi: 10.1093/advances/nmy063 PMC6363529 30721959

[171] Oelschlaeger TA. Mechanisms of Probiotic Actions - A Review. Int J Med Microbiol (2010) 300(1):57–62. doi: 10.1016/j.ijmm.2009.08.005 19783474

[172] Laskowska E Jarosz L Gradzki Z. Effect of Multi-Microbial Probiotic Formulation Bokashi on Pro- and Anti-Inflammatory Cytokines Profile in the Serum, Colostrum and Milk of Sows, and in a Culture of Polymorphonuclear Cells Isolated From Colostrum. Probiotics Antimicrob Proteins (2019) 11(1):220–32. doi: 10.1007/s12602-017-9380-9 PMC6449489 29305686

[173] Khorasani S Mahmoudi M Kalantari MR Lavi Arab F Esmaeili SA Mardani F. Amelioration of Regulatory T Cells by Lactobacillus Delbrueckii and Lactobacillus Rhamnosus in Pristane-Induced Lupus Mice Model. J Cell Physiol (2019) 234(6):9778–86. doi: 10.1002/jcp.27663 30370554

[174] Hsu TC Huang CY Liu CH Hsu KC Chen YH Tzang BS. Lactobacillus Paracasei GMNL-32, Lactobacillus Reuteri GMNL-89 and L. Reuteri GMNL-263 Ameliorate Hepatic Injuries in Lupus-Prone Mice. Br J Nutr (2017) 117(8):1066–74. doi: 10.1017/S0007114517001039 28502277

[175] Mennigen R Bruewer M. Effect of Probiotics on Intestinal Barrier Function. Ann N Y Acad Sci (2009) 1165:183–9. doi: 10.1111/j.1749-6632.2009.04059.x 19538305

[176] Mu Q Zhang H Liao X Lin K Liu H Edwards MR. Control of Lupus Nephritis by Changes of Gut Microbiota. Microbiome (2017) 5(1):73. doi: 10.1186/s40168-017-0300-8 28697806 PMC5505136

